# Disilane Cleavage with Selected Alkali and Alkaline Earth Metal Salts

**DOI:** 10.1002/chem.201902722

**Published:** 2019-09-13

**Authors:** Tobias Santowski, Alexander G. Sturm, Kenrick M. Lewis, Thorsten Felder, Max C. Holthausen, Norbert Auner

**Affiliations:** ^1^ Institut für Anorganische und Analytische Chemie Goethe-Universität Max-von-Laue-Straße 7 60438 Frankfurt/Main Germany; ^2^ Momentive Performance Materials Inc. 769 Old Saw Mill River Rd Tarrytown NY 10591 USA; ^3^ Momentive Performance Materials GmbH, Chempark 51368 Leverkusen Germany

**Keywords:** alkali and alkaline earth metal salts, disilane cleavage, lithium chloride, lithium hydride, monosilanes

## Abstract

The industry‐scale production of methylchloromonosilanes in the Müller–Rochow Direct Process is accompanied by the formation of a residue, the direct process residue (DPR), comprised of disilanes Me_*n*_Si_2_Cl_6‐*n*_ (*n*=1–6). Great research efforts have been devoted to the recycling of these disilanes into monosilanes to allow reintroduction into the siloxane production chain. In this work, disilane cleavage by using alkali and alkaline earth metal salts is reported. The reaction with metal hydrides, in particular lithium hydride (LiH), leads to efficient reduction of chlorine containing disilanes but also induces disproportionation into mono‐ and oligosilanes. Alkali and alkaline earth chlorides, formed in the course of the reduction, specifically induce disproportionation of highly chlorinated disilanes, whereas highly methylated disilanes (*n*>3) remain unreacted. Nearly quantitative DPR conversion into monosilanes was achieved by using concentrated HCl/ether solutions in the presence of lithium chloride.

## Graphical abstract

**Too valuable for disposal or incineration**: Simple recycling of the Müller–Rochow Direct Process residue with LiH yields monosilanes suitable for the reintroduction into the silicone production chain.

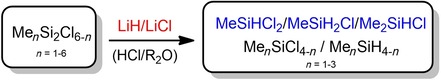

## Introduction

Methylchlorosilanes Me_*n*_SiCl_4‐*n*_ (*n*=1–3) are produced in the Direct Process (DP)^1^ at large scales by reaction of elemental silicon with chloromethane. The production of the main product Me_2_SiCl_2_,^2^ however, is accompanied by the formation of an unwanted residue (the DPR) comprised of methylchlorodisilanes, Me_*n*_Si_2_Cl_6‐*n*_ (*n*=1–6) and, in minor amounts, of carbodisilanes, which accumulate in tens of thousands of tons annually.^3^ Owing to the fact that enormous amounts of silicon are consumed for DPR formation, great efforts have been made in the past to devise preparative protocols for the conversion of the DPR into the corresponding monosilanes.3a, 4 Specifically, the Lewis‐base induced disproportionation of disilanes under moderate reaction conditions and disilane splitting can be achieved by use of catalytic amounts of phosphines,^5^ amines,^6^ as well as phosphonium or ammonium chlorides.^7^ These reactions afford transformation of the DPR constituents into silane monomers along with oligo‐ or polysilanes as side products.^8^ Based on suggestions put forth in the literature6a, 9 the course of disilane cleavage involves nucleophilic attack of the Lewis base at the disilane moiety with subsequent extrusion of a Lewis‐base‐stabilized silylene and formation of a monosilane. Silylene insertion into a second disilane equivalent gives rise to trisilane formation. Reiteration of this step results in high molecular weight, in some cases insoluble, polysilanes.9b To reduce oligosilane formation, hydrogen chloride (HCl) is usually added as in situ trapping agent for the silylenes formed.^10^ In any case, the workup of these oligosilanes, or their disposal by incineration, reduces the economic benefit of the overall DP significantly.

Recently, we have reported on the competitive chlorination and cleavage of methylhydridodisilanes with ether/HCl solutions to yield bifunctional monosilanes in excellent yields.^11^ The methylhydridodisilanes used there were synthesized by reduction of the corresponding chloro‐substituted precursors with LiAlH_4_ as the hydrogenation agent. In search of alternative hydride sources, we reacted methylchlorodisilane mixtures mimicking the DPR with lithium hydride. To our surprise, we found that most disilanes are efficiently cleaved, resulting in formation of mostly bifunctional monosilanes in high yields (see below). These findings prompted us to conduct further investigations on the disilane hydrogenation and cleavage reactions with alkali‐ and alkaline earth hydrides. The results of these studies are reported in the following.

A number of earlier studies by others have shown that the hydrogenation of chlorosilanes can efficiently be achieved by using complex reducing agents, such as LiAlH_4_,^12^ NaBH_4_ 13 and LiBH_4_.^14^ Also the alkali metal hydrides LiH^15^ and NaH^16^ as well as alkaline earth metal hydrides such as MgH_2_ 17 have been used for chlorosilane reduction. Polyether solvents have been often used to activate LiBH_4_ and NaBH_4_ for reduction of, for example, SiCl_4_, Me_2_SiCl_2_ or GeCl_4_.^18^ Chlorosilane reductions are generally performed at ambient temperatures (20–25 °C) as temperatures above 100 °C often cause decomposition of the reducing agents or the desired product.^18^ Moreover, calcium and titanium hydrides,^19^ or mixtures of NaH/NaBH_4_ 20 have been found effective in reduction reactions, but all synthetic routes reported thus far are lacking selectivity and yield the perhydrido‐substituted derivatives as main products.

Bifunctional monosilanes represent fundamentally important building blocks in silicone technology. Utilizing 1) the Si−H functionality for hydrosilylation reactions to create silicon–carbon bonds^21^ and 2) the Si−Cl functions for hydrolysis or alcoholysis provides access to the corresponding silanols or alkoxysilanes employed in condensation reactions to form the siloxane Si−O−Si bonding motif.4k, 22 For the synthesis of bifunctional monosilanes, some preparative protocols have been reported: As shown by D′Errico and Sharp for a variety of halosilanes, the selective reduction of a single Si−Cl bond is possible by using alkyltin hydrides.^23^ Further, the Roewer group converted Me_2_SiCl_2_ to Me_2_SiHCl with organotin hydrides in the presence of phosphonium chlorides or amine bases.^24^ More recently, Ir‐mediated synthetic protocols utilizing H_2_ as hydrogen source have been reported.^25^ Alternatively, efficient access to monosilanes R_2_SiHCl and RSiHCl_2_ has been established by selective chlorination of hydridomonosilanes with HCl in the presence of catalytic amounts of Lewis acids^26^ or Lewis‐bases such as ethers.^27^

The efficient cleavage of silicon‐silicon bonds with alkali metal salts has been first reported by Ring and co‐workers.^28^ This group studied reactions of Si_2_H_6_ with alkali metal chlorides and hydrides to yield SiH_4_, ‐(SiH_2_)_*n*_‐ polymers and metal silanides,^29^ and also the cleavage of some alkyldisilanes was investigated.^30^ Furthermore, the pertinent patent literature reports on metal‐salt‐catalyzed cleavage of different disilanes present in the DPR and disclosed alkali metal halides to form complexes with various tertiary amines, which are effective in cleavage reactions.^31^ We here report the cleavage reactions of different methylchlorodisilanes with alkali and alkaline earth metal salts to give monosilanes in high yields. We focus in particular on the synthesis of bifunctional monosilanes, bearing both hydrido and chloro substituents, formed by simultaneous cleavage and reduction of the disilanes present in the DPR.^28^, ^29^, ^30^

## Results and Discussion

Highly chlorinated disilanes such as Cl_2_MeSi−SiMeCl_2_ (**1**, 50–75 wt % of the DPR) and ClMe_2_Si−SiMeCl_2_ (**2**, 20–40 wt %) are referred to in the literature as “cleavable fraction” of the DPR, because they can be thermally cleaved by amine catalyzed reaction with HCl.10a In contrast, the term “uncleavable fraction” has been coined for the highly methylated disilanes ClMe_2_Si−SiMe_2_Cl (**3**, 4–10 wt %), Me_3_Si−SiMeCl_2_ (**4**, 2–4 wt %), Me_3_Si−SiMe_2_Cl (**5**, 3–6 wt %) and Me_3_Si−SiMe_3_ (**6**, 0–1 wt %) as their cleavage requires harsh conditions.3b, 3c, 4a, 4c, 32 Only recently cleavage of these disilanes has been reported, but yields of bifunctional monosilanes were low.4a In principle, the “uncleavable” fraction of the DPR can be transformed into “cleavables” by high temperature Si−Cl/Si−Me redistribution reactions with HCl in the presence of AlCl_3_,^33^ but clearly this process is cumbersome.

Table 1 lists the numbering scheme of starting materials and products relevant in this study, procedures as well as NMR spectroscopic data are provided as Supporting Information. Disilanes were separated from authentic industrial DPR samples and hydridodisilanes and hydridocarbodisilanes were obtained by reduction of chlorinated precursors with LiAlH_4_ maintaining the Si−Si and the Si−C−Si backbone.

**Table 1:** Numbering scheme of silanes reacted and reaction products formed.

No.	Compounds	No.	Compounds	No.	Compounds
**1**	Cl_2_MeSi−SiMeCl_2_	**14**	Me_3_SiCl	**27**	Cl_2_MeSi−SiMe_2_H
**2**	ClMe_2_Si−SiMeCl_2_	**15**	Me_3_SiH	**28**	ClMe_2_Si−SiMeH_2_
**3**	ClMe_2_Si−SiMe_2_Cl	**16**	H_2_MeSi−SiMeH_2_	**29**	HMe_2_Si−SiMeClH
**4**	Me_3_Si−SiMeCl_2_	**17**	HMe_2_Si−SiMeH_2_	**30**	Cl_2_MeSi−CH_2_−SiMeCl_2_
**5**	Me_3_Si−SiMe_2_Cl	**18**	HMe_2_Si−SiMe_2_H	**31**	ClMe_2_Si−CH_2_−SiMeCl_2_
**6**	Me_3_Si−SiMe_3_	**19**	Me_3_Si−SiMeH_2_	**32**	ClMe_2_Si−CH_2_−SiMe_2_Cl
**7**	MeSiCl_3_	**20**	Me_3_Si−SiMe_2_H	**33**	Me_3_Si−CH_2_−SiMeCl_2_
**8**	MeSiHCl_2_	**21**	ClMe_2_Si−SiMe_2_H	**34**	Me_3_Si−CH_2_−SiMe_2_Cl
**9**	MeSiH_2_Cl	**22**	Cl_2_MeSi−SiMeClH	**35**	Me_3_Si−CH_2_−SiMe_3_
**10**	MeSiH_3_	**23**	HClMeSi−SiMeClH	**36**	Me_3_Si−CH_2_−SiMe_2_H
**11**	Me_2_SiCl_2_	**24**	Cl_2_MeSi−SiMeH_2_	**37**	HMe_2_Si−CH_2_−SiMe_2_H
**12**	Me_2_SiHCl	**25**	HClMeSi−SiMeH_2_	**38**	HMe_2_Si−CH_2_−SiMeH_2_
**13**	Me_2_SiH_2_	**26**	ClMe_2_Si−SiMeClH	**39**	H_2_MeSi−CH_2_−SiMeH_2_

To study the reduction and cleavage of disilanes with lithium hydride, we chose tetramethyldichlorodisilane (**3**) as a representative model compound for the “uncleavable” fraction of the DPR. **3** was quantitatively reduced at room temperature (RT) to tetramethyldisilane **18** with two equivalents of lithium hydride [Eq. (1)].

(1) $$\mathrm{ClMe}{}_{2}\mathrm{Si}-\mathrm{SiMe}{}_{2}\mathrm{Cl}+2\;\mathrm{LiH}\to \mathrm{HMe}{}_{2}\mathrm{Si}-\mathrm{SiMe}{}_{2}H+2\;\mathrm{LiCl}$$

Increase of the reaction temperature to 140 °C with excess of LiH led to cleavage of **18** to form dimethylsilane (94 %) together with the NMR‐detectable oligosilanes HMe_2_Si−(SiMe_2_)_*n*_‐SiMe_2_H (*n*=1–3, 6 %, cf. the Supporting Information).^34^ In contrast to the mechanistic picture recently established,^35^ we found that lithium chloride does not afford disilane cleavage: in a set of NMR experiments, no reaction with lithium chloride was observed for disilanes **16** 36 and **18** even at elevated temperatures. Also in contrast to earlier reports,28a, 30 both disilanes were efficiently cleaved into silane monomers with lithium hydride. Already at RT the reaction with **16** afforded MeSiH_3_ (44 %) along with oligosilanes (4 %), 52 % of disilane **16** remained uncleaved. Upon increasing the reaction temperature to 60 °C, only MeSiH_3_ was identified by ^29^Si NMR spectroscopy. The reaction of **18** with excess LiH was studied further in variable‐temperature NMR experiments: Me_2_SiH_2_ was, apart from traces of oligosilanes, the only product detectable up to 140 °C.^37^

**Scheme 1:**
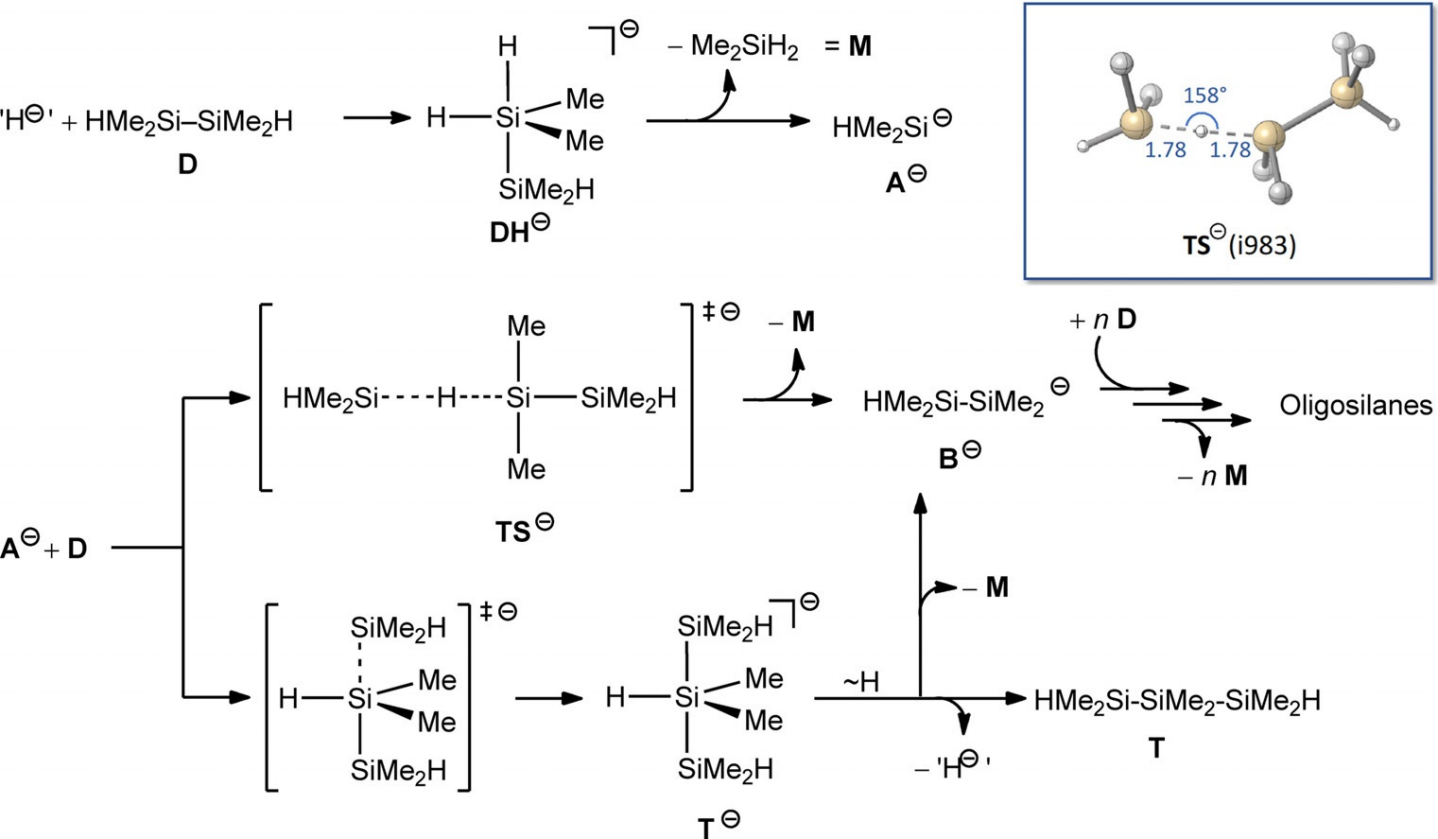
Suggested mechanism of the LiH induced formation of HMe_2_Si^−^ (A^−^) and Me_2_SiH_2_ with concomitant Aufbau of higher oligosilanes. The inset shows the transition state structure TS^−^ computed for the proton abstraction step with selected structural parameters.

Based on earlier detailed studies on the chloride‐induced aufbau of higher perchlorinated oligosilanes from Si_2_Cl_6_,^35^ we devised a tentative reaction mechanism for the cleavage of disilane **18** (Scheme 1). As the initial step, we assume the formation of a silicate **DH** ^−^ by attachment of a hydride ion, released from the LiH solid, to one of the silicon centers in disilane **18** (**D**), which subsequently undergoes Si−Si bond cleavage to give Me_2_SiH_2_ (**M**) and the silanide anion HMe_2_Si^−^ (**A** ^−^).35a, 38 The silanide **A** ^−^ can then abstract a proton^39^ from another equivalent of **D** to yield monosilane Me_2_SiH_2_ (**M**) along with the higher silanide anion HMe_2_Si−SiMe_2_^−^ (**B^−^**). A quantum chemical assessment of this step at the SMD(THF)‐M062X/6‐31+G(d,p) level reveals a moderate exoergicity (Δ_R_ *G*=−4 kcal mol^−1^) and an activation barrier of Δ^≠^ *G*=28 kcal mol^−1^ (Scheme 1), which is in line with a reaction efficiently taking place only at elevated temperature. Alternatively, **A^−^** can add to **D** to yield the higher silicate **T^−^**. This species can then either undergo hydride migration to the terminal silyl group followed by Si−Si bond cleavage to yield **M** and **B^−^**, or release a hydride ion back to the LiH solid, which results in formation of the trisilane **T**. With **T** undergoing the same reaction cascade the formation of higher oligosilanes HMe_2_Si−(SiMe_2_)_*n*_−SiMe_2_H results, which eventually become insoluble and escape NMR spectroscopic identification (for characterized species with *n*=1–4 see Supporting Information). Overall, this scenario is in line with the previous work of Ring and co‐workers^30^ who showed that disilane cleavage with lithium hydride results in monosilanes, oligosilanes and/or lithium silanides. At variance with Ring's experiments conducted at RT we do observe, however, cleavage of multiply methylated disilanes with lithium hydride at elevated reaction temperatures. We note in passing that neither **3** nor **18** react with LiCl, even at temperatures as high as 220 °C.

Much to our surprise, dimethyltetrachlorodisilane (**1**), chosen as a representative model for the “cleavable” fraction of the DPR, does not form dimethyltetrahydridodisilane **16** upon reaction with 4 equiv LiH but undergoes quantitative cleavage into monosilanes. Most notably, the industrially important bifunctional silanes MeSiHCl_2_ and MeSiH_2_Cl comprise almost 80 % of the product mixture obtained at RT. We optimized their yield by using substoichiometric amounts of LiH: The reaction with 1.3 equiv LiH yields **8** and **9** in almost 90 %, with **8** in significant excess (Table 2).

**Table 2:** Reaction products from Cl_2_MeSi−SiMeCl_2_ (**1**) and different molar amounts of LiH (mol %).

Compound	1.3 equiv LiH, RT	1.3 equiv LiH, 60 °C	2.7 equiv LiH, RT	2.7 equiv LiH, 60 °C	4.0 equiv LiH, RT	4.0 equiv LiH, 60 °C
MeSiCl_3_ (**7**)	11	1	2	–	1	–
MeSiHCl_2_ (**8**)	74	49	47	15	32	8
MeSiH_2_Cl (**9**)	15	40	40	44	46	35
MeSiH_3_ (**10**)	–	10	11	41	21	57

In contrast to our observations for **18** detailed above, the analogous reduction of disilane **1** to yield **16** is not possible with LiH. Instead, Si−Si bond cleavage interferes and **1** is quantitatively converted into monosilanes already at RT (Table 2; oligosilanes necessarily formed in this process are not NMR visible). Evidently, partial reduction of **1** has taken place already with 1.3 equiv LiH at RT giving rise to the formation of LiCl, which might trigger chloride‐induced disilane disproportionation under these conditions.^35^ This supposition was corroborated in further experiments: treatment of **1** with catalytic amounts of LiCl at RT in polar solvents, such as glymes, THF or 1,4‐dioxane, resulted in MeSiCl_3_ formation, comprising 50 % of the reaction mixture along with unreacted **1** and oligosilanes according to ^29^Si NMR analysis. Full consumption of **1** is observed at longer reaction times and higher temperatures (Table 3).34d This observation contrasts the inability of LiCl to induce cleavage of highly methylated disilane **3**; also the fully reduced dimethyldisilane **16** shows no sign of Si−Si bond cleavage in the presence of LiCl (cf. the Supporting Information).

**Table 3:** Cleavage reactions of Cl_2_MeSi−SiMeCl_2_ (**1**) with LiCl at different temperatures (mol %).

Compound	RT (30 h)	RT (60 h)	60 °C (2 h)	80 °C (2 h)	100 °C (2 h)	120 °C (2 h)	220 °C (6 h)
Cl_2_MeSi−SiMeCl_2_ (**1**)	44	28	21	5	4	2	1
MeSiCl_3_ (**7**)	50	66	69	87	91	93	96
Oligosilanes	6	6	10	8	5	5	3

We thus conclude that reaction of disilane **1** with LiH initially leads to partial reduction and kinetically favored Si−Si bond cleavage sets in, once sufficient amounts of LiCl have formed. The resulting monosilanes, in turn, are then partially reduced by LiH to yield the bifunctional monosilanes observed in the experiments. These findings complement our related study on the disilane cleavage with phosphonium chloride salts^40^ and will be addressed again in the next section.

For further scrutiny, we investigated the reaction of an authentic sample of a highly chlorinated DPR fraction, comprised of **1** (69 mol %), **2** (26 %), **3** (4 %), and **4** (1 %) dissolved in diglyme, with LiH (50 mol % with reference to the total chlorine content of the mixture). After about 30 min at 60 °C disilanes were almost quantitatively consumed and the bifunctional monosilanes MeSiH_2_Cl (33 %), MeSiHCl_2_ (21 %), and Me_2_SiHCl (9 %) were formed along with MeSiCl_3_, Me_2_SiCl_2_, and Me_3_SiCl (together 23 %) and MeSiH_3_ (13 %). As detailed in the Supporting Information, use of substoichiometric amounts of LiH leads to predominant formation of bifunctional monosilanes in up to 70 %, whereas use of 400 mol % LiH results in complete reduction to Me_2_SiH_2_ (6 %) and MeSiH_3_ (78 %) together with formation of hydridodisilanes **16**–**19** (16 %) that remained stable under the reaction conditions. Treatment of a mixture of the highly methylated chlorodisilanes **3**–**4** with different molar amounts of LiH, in turn, led to partial disilane reduction while cleavage reactions were not detected in significant amounts in most cases. Only the reaction with a high excess of LiH (350 mol %) led to 50 % disilane cleavage at 140 °C (cf. Table S10).

**Scheme 2:**
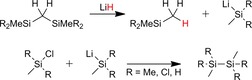
Proposed reaction pathway for the cleavage of carbodisilanes to yield disilanes that are subsequently cleaved by LiH.

Generally, the disilane fraction of the DPR is contaminated with carbodisilanes. A representative mixture of **30** (45 %), **31** (31 %), **32** (14 %), **34** (10 %) and **35** (1 %) was reacted with excess LiH (suspended in diglyme in a sealed NMR tube, cf. section 6 in the Supporting Information). Heating the sample to 180 °C led to carbodisilane reduction and Si‐C cleavage to give MeSiH_3_ (37 %) and Me_2_SiH_2_ (31 %) as main products, along with the hydridocarbodisilanes **36**–**39** (32 %).^41^ Scheme 2 illustrates a tentative mechanistic suggestion that involves initial hydride‐induced Si−C bond cleavage resulting in formation of methylsilanes together with lithium silanides. The latter undergo coupling with chlorinated monosilanes to form disilanes,^42^ which are subsequently cleaved in the presence of excess LiH.^43^

The suitability of other alkali and alkaline earth metal chlorides to induce disilane disproportionation was investigated in reactions with an industrial DPR mixture (Table S29, Supporting Information). Although disilane conversion was found most effective with LiCl in diglyme, the use of NaCl, KCl, CaCl_2_, and MgCl_2_ is impeded by their lower solubility. Acceptable reaction rates, however, were found in tetraglyme at 140 °C and above. In exemplary reactions performed at a preparative scale with both, LiCl and KCl, the DPR mixture was efficiently converted: with a maximum theoretical yield of 50 % the disproportionation led to 42 % monosilanes (predominantly MeSiCl_3_ and Me_2_SiCl_2_, cf. Supporting Information). The residue remaining after distillation of the monosilanes consists of highly methylated disilanes, carbodisilanes and oligosilanes. A broad signal at +35 ppm in the ^29^Si NMR spectrum of the sample was assigned to branched oligosilanes with terminal Cl_2_MeSi groups. Slightly higher conversion ratios were obtained with LiCl at 220 °C.^44^

## Conclusions

In summary, we have shown that chlorosilane reduction is possible with lithium hydride, which thereby is established as economically favorable alternative to LiAlH_4_. We have further shown that LiCl, formed in the course of the reduction of chlorinated disilanes with LiH, acts as an efficient catalyst to trigger disproportionation of disilanes bearing SiMeX_2_ groups (X=H, Cl) into the corresponding mono‐ and higher oligosilanes.^45^ Si−Si bond cleavage of highly methylated as well as perhydrogenated disilanes was not observed with lithium chloride. We found, however, that lithium hydride efficiently triggers disproportionation of perhydrogenated disilanes into MeSiH_3_, Me_2_SiH_2_, and Me_3_SiH and oligosilanes.

## Experimental Section

### General procedure for disilane cleavage reactions

For the elucidation of the reaction conditions, disilanes Me_*n*_Si_2_Cl_6‐*n*_ (*n*=2–6) were isolated from the DPR by fractional distillation and investigated as pure model compounds or in complex mixtures. The reactants for example, HCl/ether solutions, catalysts and solvents were placed in an NMR tube under nitrogen atmosphere and cooled to −196 °C, subsequently the disilanes were added and frozen. Then the NMR tube was evacuated (at −196 °C) and sealed in vacuo to avoid losses of low boiling monosilanes, such as MeSiH_3_ (b.p. −58 °C), Me_2_SiH_2_ (b.p. −20 °C), MeSiH_2_Cl (b.p. −46 °C), MeSiHCl_2_ (b.p. 41 °C) and Me_2_SiHCl (b.p. 35 °C). After warming the mixture to RT the reaction temperatures were increased, and the course of reaction was followed by NMR spectroscopy, especially by ^29^Si NMR. The molar ratios of products formed were determined by integration of product specific NMR signals o f the resulting mixtures. According to the optimum reaction conditions evaluated from the NMR investigations, upscaling was performed with larger amounts of starting materials in closed reaction ampules. Filling of reactants was similar as described for the experiments in sealed NMR tubes. Alternatively, upscaling was performed in open systems. This procedure is described in the Supporting Information.^40^

## Conflict of interest

The authors declare no conflict of interest.

## Supporting information

As a service to our authors and readers, this journal provides supporting information supplied by the authors. Such materials are peer reviewed and may be re‐organized for online delivery, but are not copy‐edited or typeset. Technical support issues arising from supporting information (other than missing files) should be addressed to the authors.

Supplementary
