# Supplementary material for: Disilane Cleavage with Selected Alkali and Alkaline Earth Metal Salts
Source: Chemistry. 2019 Sep 13;25(57):13202–7. doi: 10.1002/chem.201902722 (PMC6856802; doi:10.1002/chem.201902722)
Supplement: Supplementary file 1 — Supplementary [file CHEM-25-13202-s001.pdf]

CHEMISTRY
A European Journal

Supporting Information

Disilane Cleavage with Selected Alkali and Alkaline Earth Metal Salts

Tobias Santowski,[a] Alexander G. Sturm,[a] Kenrick M. Lewis,[b] Thorsten Felder,[c]
Max C. Holthausen,*[a] and Norbert Auner*[a]

chem_201902722_sm_miscellaneous_information.pdf

Supporting Information

Table of Content

1. General 3

1.1. General procedure for experiments performed in sealed NMR tubes 3
1.2. NMR spectroscopy 3
1.3. GC-MS analysis 3

2. NMR spectroscopic identification of starting materials and products 4

3. Reduction and cleavage reactions of different model compounds with lithium hydride and lithium chloride 5

3.1. Reactions of $ClMe_2Si$-$SiMe_2Cl$ (**3**) with LiH 5
3.2. Reactions of Cl_2MeSi-$SiMeCl_2$ (**1**) with LiH 5
3.3. Reactions of H_2MeSi-$SiMeH_2$ (**16**) with LiH and LiCl 5
3.4. Reactions of HMe_2Si-$SiMe_2H$ (**18**) with LiH and LiCl 6
3.5. Cleavage of HMe_2Si-$SiMe_2H$ (**18**) with LiH in a sealed ampule (preparative scale) 7
3.6. Preparation of HMe_2Si-CH_2-$SiMe_2H$ 12

4. Cleavage reactions of Cl_2MeSi-$SiMeCl_2$ (1) with LiCl 13

5. Reduction and cleavage reactions of different disilanes with alkali and alkaline earth metal hydrides 13

5.1. Reactions of highly chlorinated disilanes with LiH (50 mol%) 13
5.2. Reactions of highly chlorinated disilanes with LiH (41 mol%) 14
5.3. Reactions of highly chlorinated disilanes with different molar amounts of LiH 14
5.4. Reactions of highly methylated disilanes with different molar amounts of LiH 15
5.5. Reactions of highly chlorinated disilanes with LiH (41 and 73 mol%) 17
5.6. Reactions of a mixture of highly methylated disilanes and different carbodisilanes, representing conventionally uncleavable components of the DPR, with LiH (50 and 100 mol%) 17
5.7. Reactions of highly chlorinated disilanes with sodium hydride (50 mol%) 18
5.8. Reactions of highly chlorinated disilanes with calcium hydride (50 mol%) 18

6. Reactions of different carbodisilanes with LiH 19

7. Reduction and cleavage reactions of different disilanes with LiH in open systems 20

7.1. General 20
7.2. Reactions of a mixture of mono- and disilanes with LiH (44 mol%) 20
7.3. Reactions of a mixture of mono- and disilanes with LiH (84 mol%) 21
7.4. Reactions of a mixture of mono-, di- and carbodisilanes with LiH (43 mol%) 22

8. Cleavage of a mixture of highly chlorinated disilanes with different alkali and alkaline earth metal chlorides 24

9. Cleavage reactions of highly branched oligosilanes with LiH 26

10. Cleavage reactions of highly branched oligosilanes with LiCl 27

11. Cleavage reactions of highly chlorinated disilanes with lithium and potassium chloride in open systems 27

11.1. Disilane cleavage reactions with LiCl 27
11.2. Disilane cleavage reactions with KCl 27

12. Disilane cleavage reactions of a mixture of highly chlorinated disilanes with LiCl in the presence of a HCl/diglyme solution 29

13. Disilane cleavage reactions of a mixture consisting mainly of highly chlorinated disilanes with KCl in the presence of a HCl/diglyme solution 29

14. Computational details 30

14.1. Cartesian coordinates 30

15. References 32

16. Author Contributions 33

1. General

Chemicals were purchased from *abcr, VWR* and *Sigma Aldrich*. Lithium hydride was donated from *Albemarle*. Benzene-d_6 (C_6D_6), toluene-d_8, 1,4-dioxane, diglyme and THF were dried over Na/benzophenone and distilled prior to use.

Ether/HCl solutions were prepared passing gaseous hydrogen chloride slowly into the corresponding ether (diglyme, 1,4-dioxane) between 0 °C and 10 °C. Saturation of the ether/HCl solution was evident by the amount of HCl passing in and the amount going out of the system (bubbler). This state was maintained for 15 minutes. The HCl concentrations in ethers were determined by weighting the solutions. The obtained concentrations at 10°C: HCl in diglyme 11.2 mol/L, in 1,4-dioxane 11 mol/L. HCl concentrations of 7 mol/L in diglyme or 1,4-dioxane were achieved by dissolution of concentrated solutions with the corresponding ether.

1.1. General procedure for experiments performed in sealed NMR tubes

The reactants and catalysts, dissolved or suspended in the respective ether as solvent with some additional C_6D_6 as lock substance for NMR spectroscopic investigations, were placed in an NMR tube under nitrogen atmosphere and cooled to -196°C. Subsequently, silanes were added, the NMR tube was frozen (-196°C), evacuated and sealed under vacuo. The reactions were performed in sealed tubes to avoid any losses of low boiling monosilanes such as Me_2SiHCl (b.p.: 35°C), $MeSiHCl_2$ (b.p.: 41°C), Me_2SiH_2 (b.p.: -20°C), $MeSiH_2Cl$ (b.p.: -46°C) and $MeSiH_3$ (b.p.: -58°C).

1.2. NMR spectroscopy

NMR analyses were performed on a Bruker AV-500 spectrometer equipped with a Prodigy BBO S1 Probe. ^{29}Si NMR chemical shifts are reported relative to external tetramethylsilane (δ = 0). The molar ratios of starting materials and reaction products depicted in tables were determined by integration of compound specific signals in the ^{29}Si NMR spectra; experimental shifts for most of the compounds discussed in this paper are in accordance to previously published data.[1]

1.3. GC-MS analysis

GC-MS analyses were measured with a Thermo Scientific Trace GC Ultra coupled with an ITQ 900MS mass spectrometer. The stationary phase (*Machery-Nagel*, PERMABOND Silane) had a length of 50 m with an inner diameter of 0.32 mm. 1 µl of analyte solution was injected, 1/25 thereof was transferred onto the column with a flow rate of 1.7 mL/min carried by helium gas. The temperature of the column was first kept at 50 °C for 10 minutes. Temperature was then elevated at a rate of 20 °C/min up to 250 °C and held at that temperature for another 40 minutes. After exiting the column, substances were ionized with 70 eV and cationic fragments were measured within a range of 34 – 600 m/z (mass per charge). Product mixtures were diluted with benzene prior to the measurement.

2. NMR spectroscopic identification of starting materials and products

No.	Compounds	δ ^{29}Si [ppm]		^1J (Si-H) [Hz]		δ^1H (H-Si) [ppm]		^3J (H-H) [Hz]	
**1**	$Cl_2MeSi-SiMeCl_2$	17.5[1a]		-		-		-	
**2**	$ClMe_2Si-SiMeCl_2$	15.0[1a]	24.6[1a]	-		-		-	
**3**	$ClMe_2Si-SiMe_2Cl$	17.1[1a]		-		-		-	
**4**	$Me_3Si-SiMeCl_2$	34.0[1a]	-14.1[1a]	-		-		-	
**5**	$Me_3Si-SiMe_2Cl$	22.5[1a]	-18.6[1a]	-		-		-	
**6**	$Me_3Si-SiMe_3$	-20.1[1c]		-		-		-	
**7**	$MeSiCl_3$	12.3[1c]		-		-		-	
**8**	$MeSiHCl_2$	10.9[1c]		280.2		5.39		2.3	
**9**	$MeSiH_2Cl$	-11.4[1e]		229.4		4.55		3.6	
**10**	$MeSiH_3$	-65.5[1e]		194.1					
**11**	Me_2SiCl_2	31.5[1c]		-		-		-	
**12**	Me_2SiHCl	11.0[1c]		223.3		4.73		4.1	
**13**	Me_2SiH_2	-38.1[1c]		187.6		3.93		4.2	
**14**	Me_3SiCl	30.1[1c]		-		-		-	
**15**	Me_3SiH	-16.6[1e]		182.9		4.48		3.0	
**16**	$H_2MeSi-SiMeH_2$	-67.8[2]		185.8		-		-	
**17**	$HMe_2Si-SiMeH_2$	-39.9[2]	-66.7[2]	180.9	180.5	-		-	
**18**	$HMe_2Si-SiMe_2H$	-39.5[3]		177.5		-		-	
**19**	$Me_3Si-SiMeH_2$	-18.3[3]	-66.0[3]	-	177.7	-		-	
**20**	$Me_3Si-SiMe_2H$	-19.2[3]	-39.4[3]	-	172.5	-		-	
**21**	$ClMe_2Si-SiMe_2H$	23.0[3]	-39.0[3]	-	176.4	-		-	
**22**	$Cl_2MeSi-SiMeClH$	23.8[3]	-6.7[3]	-	227.4	-		-	
**23**	$HClMeSi-SiMeClH$	-3.9[3]	-4.3[3]	211.7		-		-	
**24**	$Cl_2MeSi-SiMeH_2$	32.1[3]	-61.4[3]	-	196.7	-		-	
**25**	$HClMeSi-SiMeH_2$	0.6[3]	-64.7[3]	215.0	203.3	-		-	
**26**	$ClMe_2Si-SiMeClH$	17.6[3]	-3.7[3]	-	221.3	-		-	
**27**	$Cl_2MeSi-SiMe_2H$	33.8[3]	-35.5[3]	-	191.3	-		-	
**28**	$ClMe_2Si-SiMeH_2$	22.6[3]	-64.6[3]	-	195.6	-		-	
**29**	$HMe_2Si-SiMeClH$	1.83	-38.2	181.3	198.4	-		-	
**30**	$Cl_2MeSi-CH_2-SiMeCl_2$	26.2[4]		-		-		-	
**31**	$ClMe_2Si-CH_2-SiMeCl_2$	28.1[4]	25.7[4]	-	-	-		-	
**32**	$ClMe_2Si-CH_2-SiMe_2Cl$	28.3[4]		-		-		-	
**33**	$Me_3Si-CH_2-SiMeCl_2$	30.6[4b]	-0.5[4b]	-	-	-		-	
**34**	$Me_3Si-CH_2-SiMe_2Cl$	30.0[4b]	-0.4[4b]	-	-	-		-	
**35**	$Me_3Si-CH_2-SiMe_3$	-0.5[1c]		-		-		-	
**36**	$Me_3Si-CH_2-SiMe_2H$	0.25[5]	-16.8[5]	-		4.0		3.6	
**37**	$HMe_2Si-CH_2-SiMe_2H$	-16.1[5]		-		4.2		3.7	
**38**	$HMe_2Si-CH_2-SiMeH_2$	-15.1[5]	-38.0[5]	-		4.1	3.9	4.0	4.3
**39**	$H_2MeSi-CH_2-SiMeH_2$	-36.5[5]		-		3.9		4.1	

3. Reduction and cleavage reactions of different model compounds with lithium hydride and lithium chloride

3.1. Reactions of $ClMe_2Si$-$SiMe_2Cl$ (3) with LiH

Tetramethyldichlorodisilane (**3**, 0.2 mL), representing a model compound of the “uncleavable” fraction of the DPR, was reacted with two equivalents of lithium hydride (17.9 mg, 100 mol%, in relation to the chlorine content of the disilane) in THF in a sealed NMR tube. Reduction started at r.t. (16 h) and tetramethyldisilane (**18**) was formed quantitatively. Performing the reaction of **3** with an excess of LiH (200mol%, in relation to the chlorine content of the disilane), **18** was formed first via reduction and subsequently cleaved at 140 °C (17 h) to quantitatively give Me_2SiH_2 (**13**) as volatile product.

3.2. Reactions of Cl_2MeSi-$SiMeCl_2$ (1) with LiH

Dimethyltetrachlorodisilane (**1**) was reacted with different molar amounts of LiH at different temperatures in THF as solvent in sealed NMR tubes. Products formed are listed in Table S1 proving the efficient reduction and cleavage of disilane **1** to form high amounts of bifunctional monosilanes already at r.t..

Table S1*

**No.**	**Compound**	**1.3 equiv. LiH r.t. (16 h)**	**1.3 equiv. LiH 60 °C (7.5 h)**	**2.7 equiv. LiH r.t. (16 h)**	**2.7 equiv. LiH 60 °C (7.5 h)**	**4.0 equiv. LiH r.t. (16 h)**	**4.0 equiv. LiH 60 °C (7.5 h)**
**7**	**$MeSiCl_3$**	11	1	2	-	1	-
**8**	**$MeSiHCl_2$**	74	49	47	15	32	8
**9**	**$MeSiH_2Cl$**	15	40	40	44	46	35
**10**	**$MeSiH_3$**	-	10	11	41	21	57

*) Values listed in the table are given in mol%.

Notably, in case dimethyldisilane (**16**, obtained from reduction of **1** with $LiAlH_4$) was used as disilane substrate for cleavage with LiH, $MeSiH_3$ (**10**) was quantitatively formed as volatile product already at 60°C.

3.3. Reactions of H_2MeSi-$SiMeH_2$ (16) with LiH and LiCl

Dimethyldisilane (**16**) (obtained from reduction of the chlorinated precursor **1** with $LiAlH_4$) was reacted with catalytic amounts of LiH at different temperatures in THF as solvent in a sealed NMR tube. Silane monomer formation ($MeSiH_3$, **10**, 44 %) started already at r.t. (16 h), simultaneously giving 4 % of oligosilanes, and was completed at 60 °C (7.5 h), as indicated by ^{29}Si NMR spectroscopy. Notably, with lithium chloride no cleavage of the silicon-silicon bond occurred, even not at 220 °C.

3.4. Reactions of $HMe_2Si\text{-}SiMe_2H$ (18) with LiH and LiCl

Tetramethyldisilane (**18**) (obtained from reduction of the chlorinated precursor **2** with $LiAlH_4$, or LiH) was reacted with LiH at different temperatures in THF as solvent in a sealed NMR tube. Silane monomer formation (Me_2SiH_2, **13**, 94 %) started at 140 °C (7.5 h) and was completed at 160 °C (16 h), as indicated by ^{29}Si NMR spectroscopy. At a reaction temperature of about 140 °C oligosilanes were detected in 6 % in the corresponding ^{29}Si NMR spectrum of the sample. These oligosilanes were identified as tri-, tetra- and pentasilanes ($HMe_2Si\text{-}(SiMe_2)_n\text{-}SiMe_2H$ (n=1-3) with $\delta^{29}Si(n=1)$[1d]: -36.7, -45.2 ppm; $\delta^{29}Si(n=2)$[6]: -36.8, -43.8 ppm; $\delta^{29}Si(n=3)$[7]: -36.9, -41.5, -46.5 ppm). Notably, with lithium chloride no cleavage of the silicon-silicon bond occurred, even not at 220 °C. With lithium hydride, a new product was observed at 160 °C which was identified as Me_3SiH. Increasing the temperature to 180 °C (17 h), the molar amount of Me_3SiH was further increased yielding Me_2SiH_2 in 84 %, Me_3SiH (14 %) and detectable oligosilanes (2%). Further heating of the sample to 200 C (21 h) proved that no oligosilanes were detected in the ^{29}Si NMR spectrum of the sample but Me_3SiH was formed as main product (59 %) besides Me_2SiH_2 (41 %). The stacked ^{29}Si NMR of the samples reacted at r.t. and after heating to 140, 160 and 200 C are shown in Figure S1.

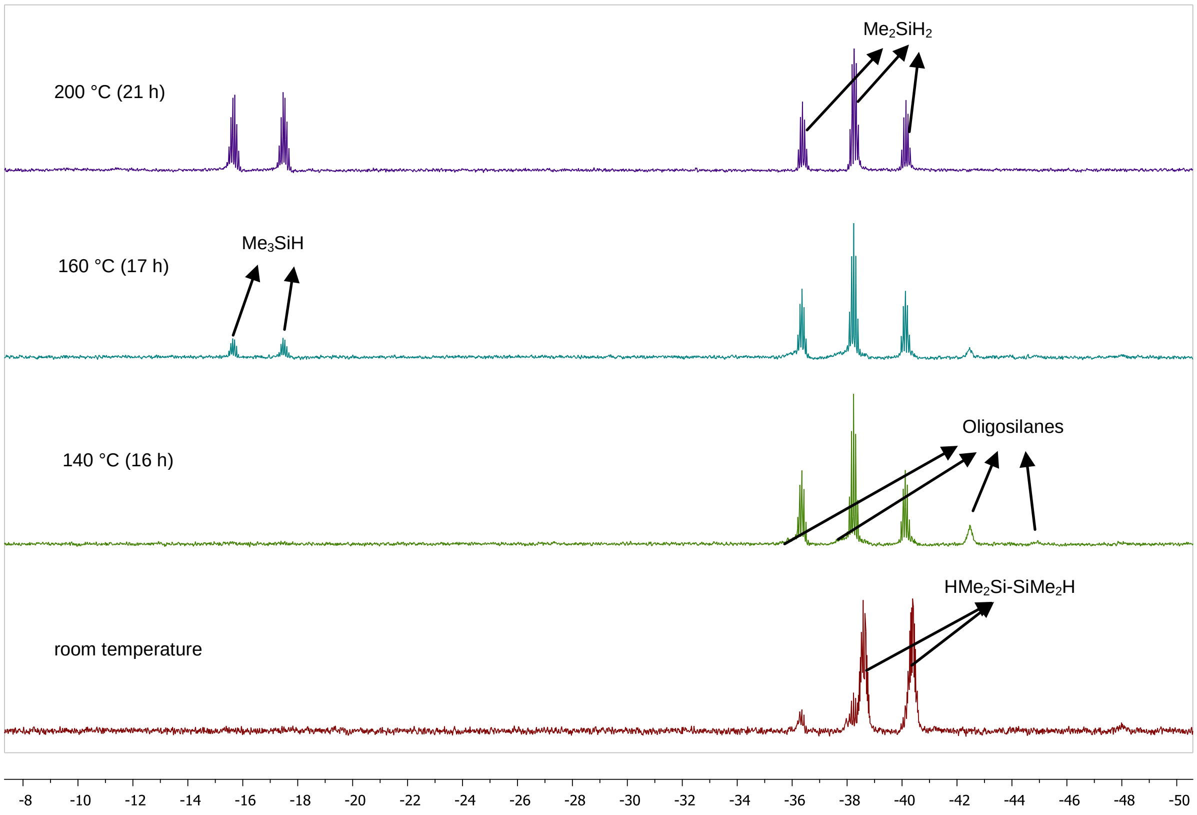

Figure S1: ^{29}Si NMR spectra of a sample representing cleavage reactions of disilane **18** with LiH at different temperatures.

3.5. Cleavage of HMe_2Si-$SiMe_2H$ (18) with LiH in a sealed ampule (preparative scale)

LiH (56 mg, 0.8 mmol, 1.3 mol%), THF (5.3 mL) and disilane **18** (4.5 g, 38.4 mmol) were placed in an ampule. Subsequently the ampule was cooled to -196 °C, evacuated and sealed. The reaction mixture was heated to 220 °C (13 h). After cooling back to r.t., the ampule was opened under nitrogen atmosphere and all volatile products formed were condensed into a Schlenk-flask in vacuo, warming the sample to a final temperature of about 250 °C. Combined condensation and distillation procedures resulted in the isolation of four fractions (a-d), traces of carbodisilanes could not been detected NMR spectroscopically, but were identified by GC-MS (see b):

a) The low boiling products Me_2SiH_2, Me_3SiH and the oligosilanes were condensed at r.t. from the Schlenk-flask into an ampule with an attached NMR tube. ^{29}Si NMR analysis gave a product composition listed in Table S2 (2.1 g). Oligosilanes were detected in traces in the corresponding ^{29}Si NMR spectrum of the sample (Figure S2).

Table S2

No.	Compound	mol%	Weight [g]	Amount of Si [mmol]
**13**	Me_2SiH_2	78	1.5	33
**15**	Me_3SiH	16	0.4	
**10**	$MeSiH_3$	1	<0.1	
**18**	HMe_2Si-$SiMe_2H$	4	0.2	
**n=1-3**	HMe_2Si-$(SiMe_2)_n$-$SiMe_2H$	traces	traces	

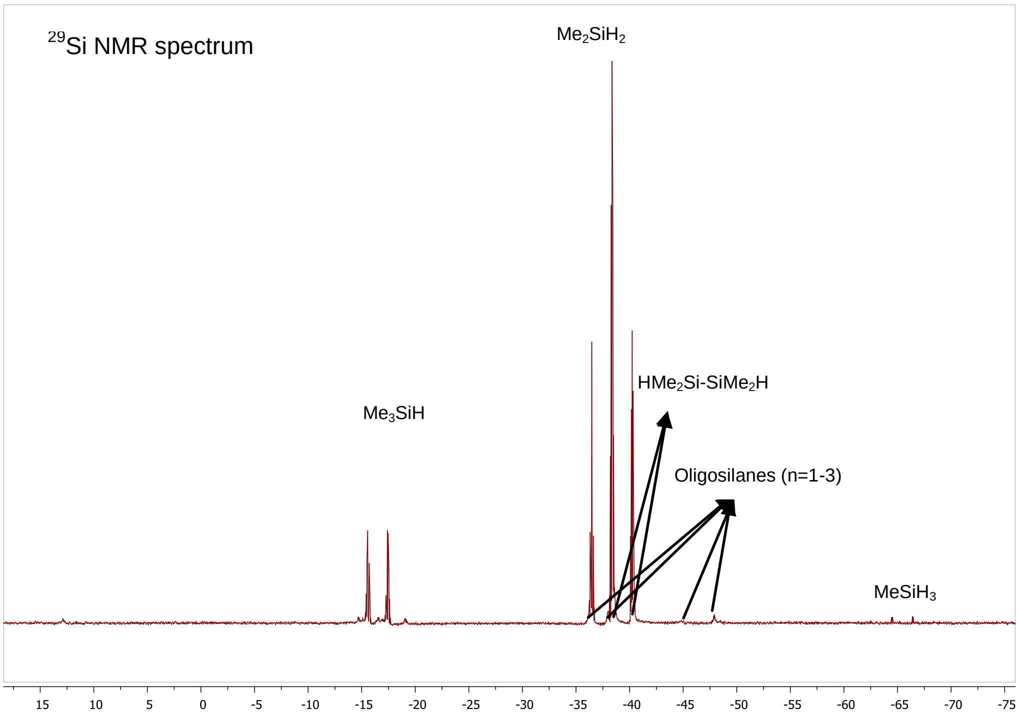

Figure S2: ^{29}Si NMR spectrum of the product mixture depicted in Table S2, a).

b) From the mixture isolated in a), Me_2SiH_2 and Me_3SiH were evaporated at r.t. The remaining residue comprises small amounts of oligosilanes HMe_2Si-$(SiMe_2)_n$-$SiMe_2H$ (n=1-3) and carbodisilane $((HMe_2Si)_2$-CH_2 as detected by GC-MS analysis (Figure S3, Table S3) of the sample.

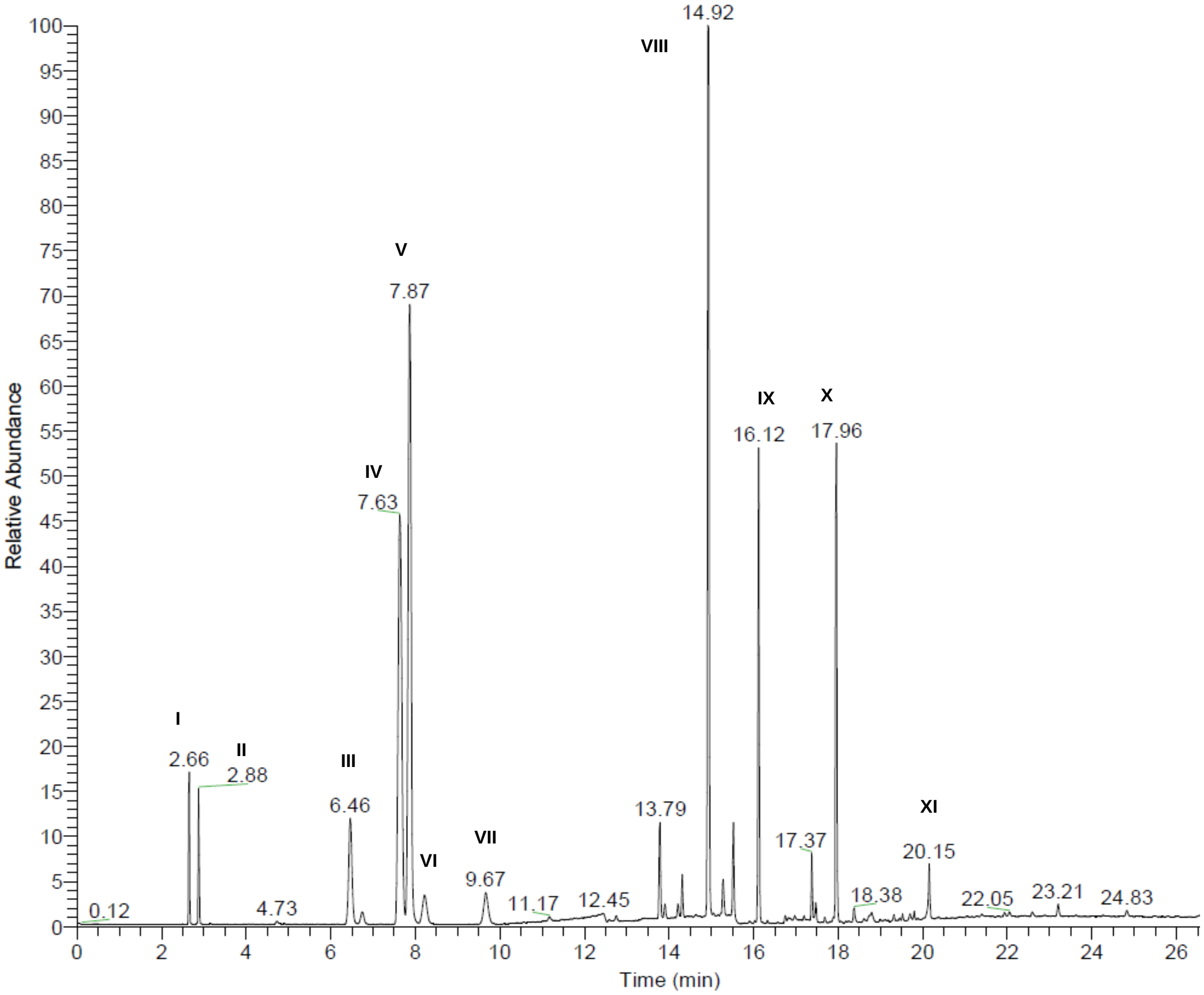

Figure S3: Gas chromatogram of the volatile product mixture b) after evaporation of most Me_2SiH_2 (**I**) and Me_3SiH (**II**), b).

Table S3: Mass spectra of silane compounds

Compound	Mass spectrum
Me_2SiH_2 **I** 59 $[M-H]^+$	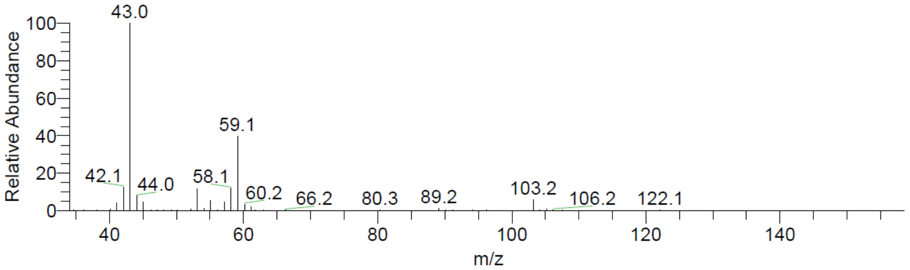
Me_3SiH **II** 73 $[M-H]^+$	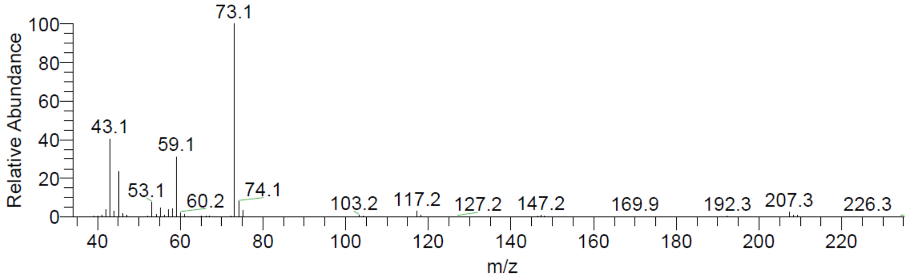

$HMe_2Si\text{-}SiMe_2H$<br>**III**<br>117 $[M\text{-}H]^+$	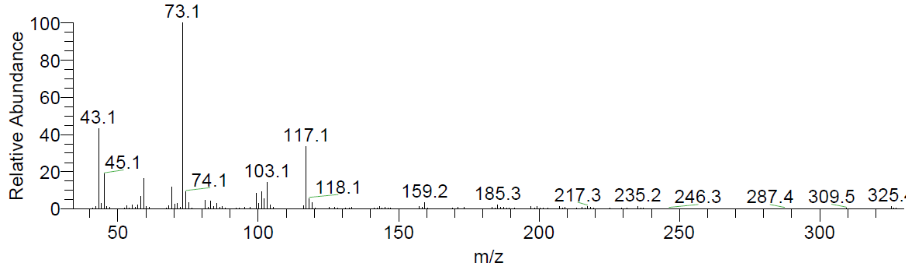
THF **IV**	71 $[M\text{-}H]^+$
C_6D_6 **V**	84 $[M]^+$
$Me_3Si\text{-}SiMe_2H$<br>**VI**<br>131 $[M\text{-}H]^+$	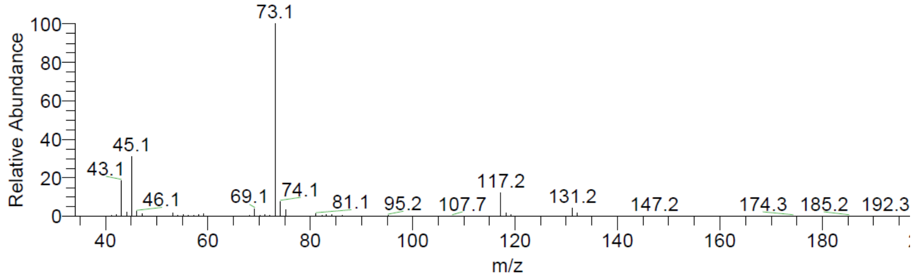
$(HMe_2Si)_2\text{-}CH_2$<br>**VII**<br>131 $[M\text{-}H]^+$	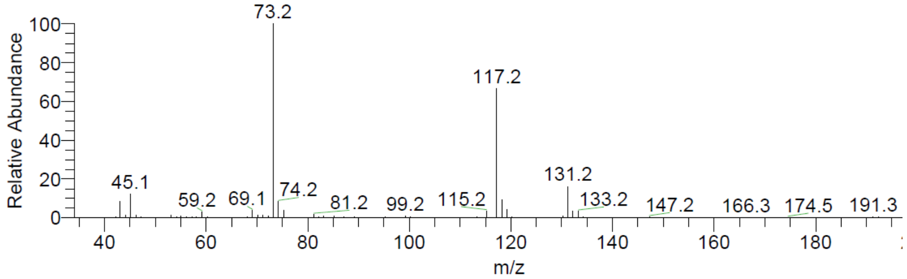
$HMe_2Si\text{-}SiMe_2\text{-}SiMe_2H$<br>**VIII**<br>175 $[M\text{-}H]^+$	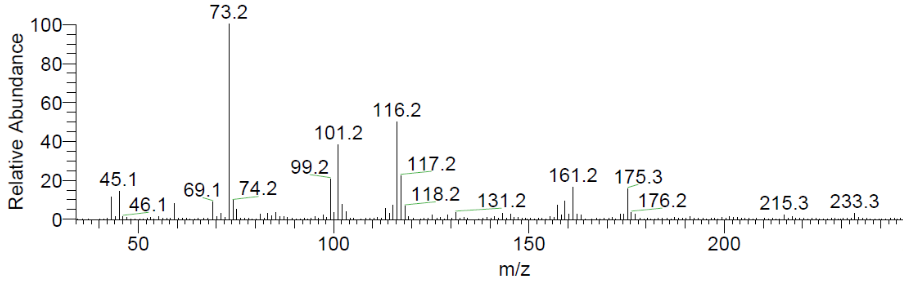
$(HMe_2Si)_3\text{-}CH$<br>**IX**<br>189 $[M\text{-}H]^+$	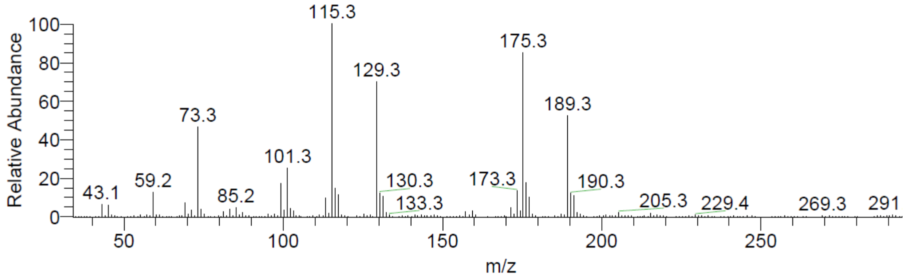
$HMe_2Si\text{-}(SiMe_2)_2\text{-}SiMe_2H$<br>**X**<br>233 $[M\text{-}H]^+$	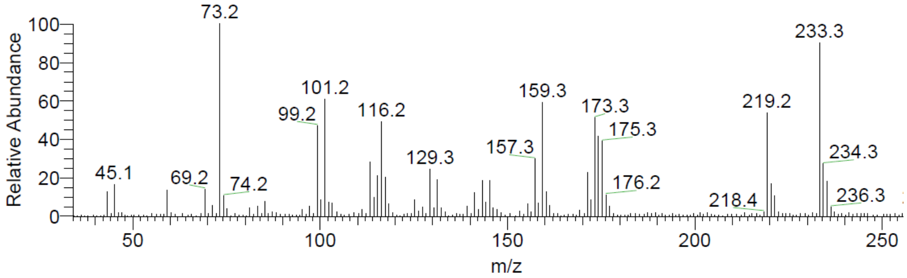

HMe_2Si-$(SiMe_2)_3$-$SiMe_2H$ **XI** 291 $[M-H]^+$	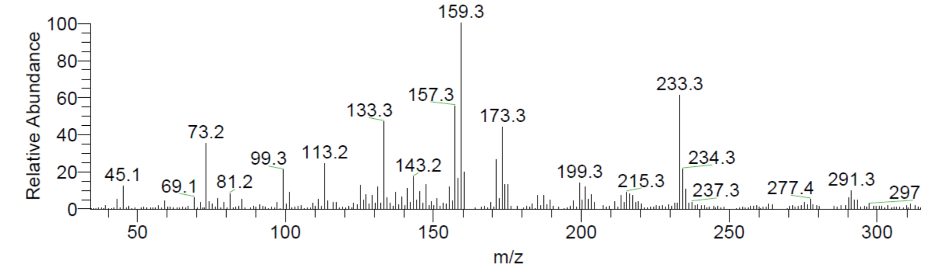
$(HMe_2Si)_4$-C **XII** 247 $[M-H]^+$	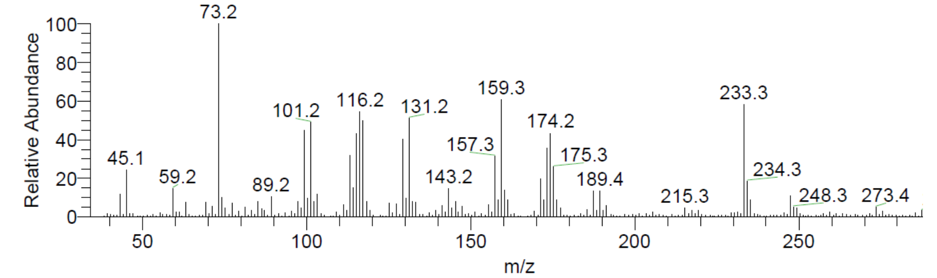
HMe_2Si-$(SiMe_2)_4$-$SiMe_2H$ **XIII** 349 $[M-H]^+$	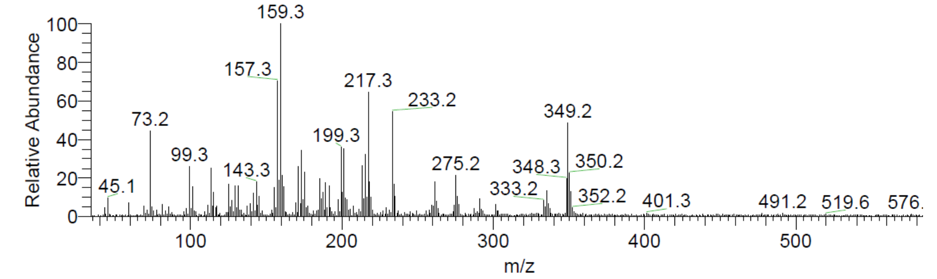

c) The remaining residue of the Schlenk-flask (Step a), 200 mg) comprises of the oligosilanes HMe_2Si-$(SiMe_2)_n$-$SiMe_2H$ (n=1-3) as well as the carbosilanes $(HMe_2Si)_3CH$ and $(HMe_2Si)_4C$ (Figure S4 and S5, see Table S4 for details, fraction 3).

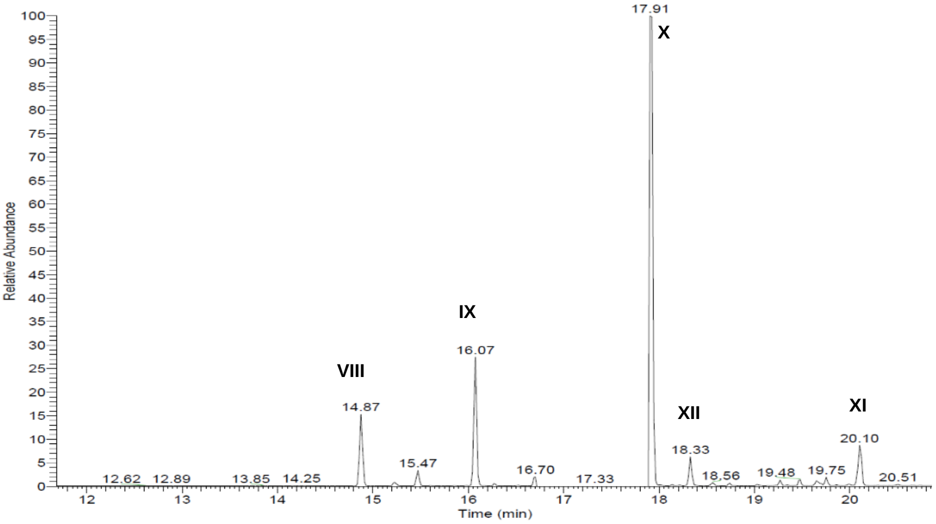

Figure S4: Gas chromatogram of the residue after separation of the volatiles in vacuo, c).

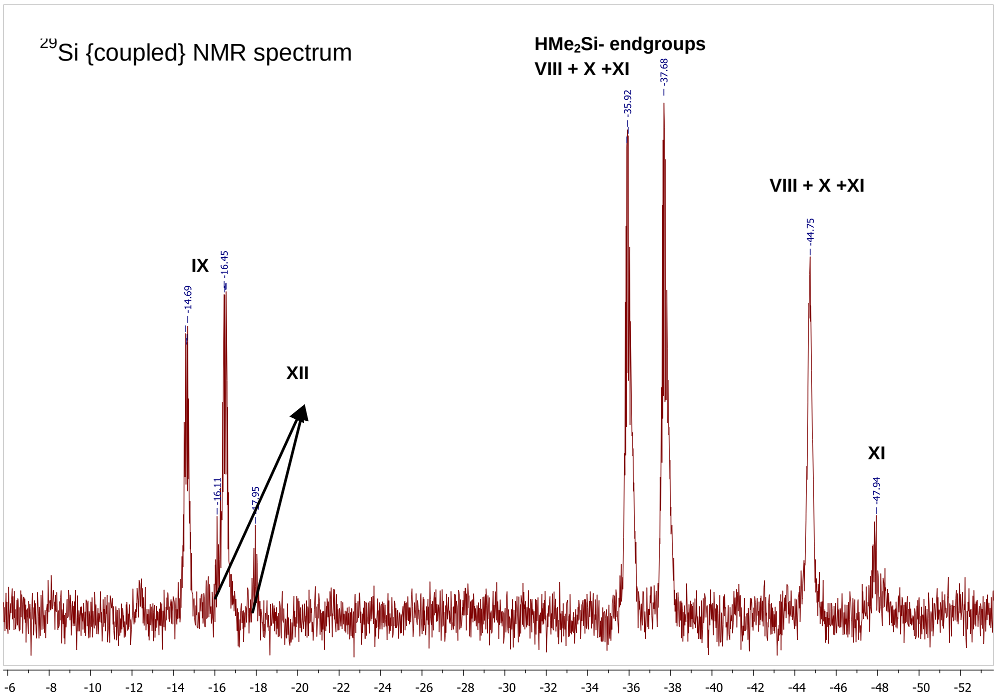

Figure S5: ^{29}Si NMR spectrum (coupled) of the residue after separation of the volatiles in vacuo, c).

Table S4

No	Compound	mol%	Weight [mg]	Amount of Si [mmol]
**VIII**	HMe_2Si-$SiMe_2$-$SiMe_2H$	9	14	3
**IX**	$(HMe_2Si)_3$-CH	16	27	
**X**	HMe_2Si-$(SiMe_2)_2$-$SiMe_2H$	65	135	
**XI**	HMe_2Si-$(SiMe_2)_3$-$SiMe_2H$	6	9	
**XII**	$(HMe_2Si)_4$-C	4	15	

d) The non-distillable residue in the starting reaction ampule and obtained after separation of volatiles at 250 °C/vacuo, (2.5 g, white to greyish solid suspended in a colorless liquid) was analyzed by GC-MS and NMR spectroscopy, indicating that the oligosilane HMe_2Si-$(SiMe_2)_n$-$SiMe_2H$ (n=4, **XI**) as well as the carbosilanes $(HMe_2Si)_3CH$ and $(HMe_2Si)_4C$ (Figure S6, Table S5) have been formed. The published NMR chemical shift for the HMe_2Si-group in carbosilanes ($^{29}\delta Si$ = -15.4 for $(HMe_2Si)_3$-CH) as well as MS data are fully in line with our analytical results.[8]

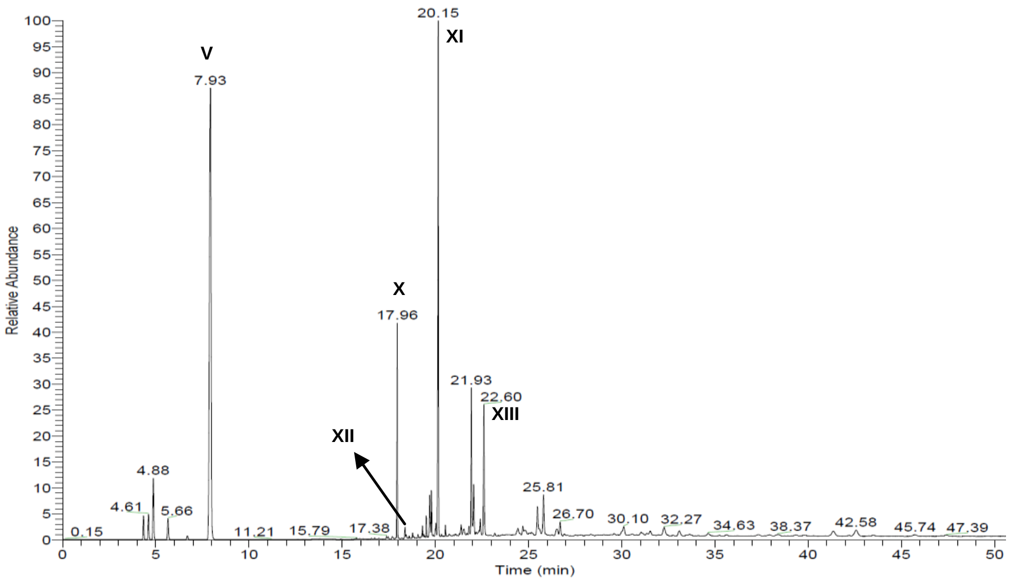

Figure S6: Gas chromatogram of the high boiling residue d).

Table S5

No	Compound	mol%	Weight [g]	Molar amount [mmol]	Amount of Si [mmol]
**X**	HMe_2Si-$(SiMe_2)_2$-$SiMe_2H$	20	0.4	1.7	42
**XI**	HMe_2Si-$(SiMe_2)_3$-$SiMe_2H$	59	1.5	0.2	
**XII**	$(HMe_2Si)_4$-C	2	<0.1	5.1	
**XIII**	HMe_2Si-$(SiMe_2)_4$-$SiMe_2H$	19	0.6	1.6	

Summary: From 4.5 g of disilane **18** we obtained 1.5 g of Me_2SiH_2 and 0.4 g Me_3SiH as silane monomers (in sum: 1.9 g, fraction a): 33 mmol Si). The HMe_2Si-substituted oligosilanes described in c) count to about 200 mg with the main component HMe_2Si-$(SiMe_2)_2$-$SiMe_2H$ (fraction c): 3 mmol Si). The non-distillable residue obtained in d) (2.5 g) consists of mainly high boiling oligosilanes such as HMe_2Si-$(SiMe_2)_3$-$SiMe_2H$ and HMe_2Si-$(SiMe_2)_4$-$SiMe_2H$ (fraction d): 42 mmol Si). The overall-yield of monosilanes (100 % = 76.4 mmol) obtained from disilane cleavage is 45 %. Other side products formed (55 %) are found to be oligosilanes as well as carbosilanes. For comparison and support of analytical identification, the carbodisilane HMe_2Si-CH_2-$SiMe_2H$ was prepared as model compounds reacting HMe_2Si-CH_2MgCl with Me_2SiHCl (See 3.6.). NMR and MS data are given below and are in full agreement with the assignments given for products listed in Tables S2-S5.

3.6. Preparation of HMe_2Si-CH_2-$SiMe_2H$

A solution of HMe_2Si-CH_2Cl (2.8 mL, 23.0 mmol, 1 eq, prepared from $LiAlH_4$ reduction of $ClMe_2Si$-CH_2Cl) in 5 mL of dry THF was added dropwise to a vigorously stirred suspension of Mg-turnings (0.78 g, 32.2 mmol, 1.4 eq, activated with $Br_2C_2H_4$, 0.05 mL) in 5 mL of dry THF. The reaction mixture was heated to reflux for 1 h and then cooled to r.t.. Excess of Mg was filtered off and the solid was washed three times with 2.5 mL of dry THF. The filtrate was cooled to 0 °C and Me_2SiHCl (2.8 mL, 25.6 mmol, 1.11 eq) was slowly added via a dropping funnel ($MgCl_2$ precipitated immediately). The reaction mixture was stirred for two days at r.t. and was then separated from $MgCl_2$ by filtration. The solid was washed

four times with 2 mL of dry THF. Fractional distillation gave 0.3 g of the desired product HMe_2Si-CH_2-$SiMe_2H$ in 10 % yield, b.p. .66 °C.

^{1}H NMR (300 MHz, C_6D_6): δ= 4.16 (sept., 2H, $^{3}J_{HH}$= 3.65 Hz, (**H**-$SiMe_2)_2$-CH_2), 0.08 (d, 12H, $^{3}J_{HH}$= 3.68 Hz, (H-Si**Me**$_2)_2$-CH_2), -0.32(t, 2H, $^{3}J_{HH}$= 3.80 Hz, (H-$SiMe_2)_2$-C**H**$_2$) ppm.

^{29}Si NMR (59.6 MHz, C_6D_6): δ=-16.1 (d, $^{1}J_{SiH}$= 182.9 Hz) ppm.

Mass spectrum: 131 $[M\text{-}H]^+$, 117 $[M\text{-}CH_3]^+$ and 73 $[Me_3Si]^+$, calculated: $[M]^+$=132

4. Cleavage reactions of Cl_2MeSi-$SiMeCl_2$ (1) with LiCl

Disilane **1** (0.15 mL) dissolved in 0.1 mL C_6D_6 and 0.4 mL diglyme was reacted with 90 mg LiCl in a sealed NMR tube. The cleavage reactions were monitored by ^{29}Si NMR spectroscopy at different temperatures and reaction times. At r.t. (90 h), 28 mol% of disilane **1** remained uncleaved. Different oligosilanes were formed besides 66 mol% of monosilane **7**. Further heating of the sample proved the cleavage reactions almost completed at 140 °C to give 93 mol% of monosilane **7**. Heating of the sample to higher temperatures showed no significant change in the product distribution. Products formed upon heating of the sample are listed in Table S6.

Table S6

No.	Compound	r.t. 30 h	r.t. 60 h	60°C 2 h	80°C 2 h	100°C 2 h	120°C 2 h	220°C 6h
**1**	**Cl_2MeSi-$SiMeCl_2$**	44	28	21	5	4	2	1
**7**	**$MeSiCl_3$**	50	66	69	87	91	93	96
	**Oligosilanes**	6	6	10	8	5	5	3

5. Reduction and cleavage reactions of different disilanes with alkali and alkaline earth metal hydrides

5.1. Reactions of highly chlorinated disilanes with LiH (50 mol%)

A mixture of highly chlorinated disilanes, Cl_2MeSi-$SiMeCl_2$ (**1**, 69 mol%), Cl_2MeSi-$SiMe_2Cl$ (**2**, 26 mol%) admixed with Cl_2MeSi-$SiMe_3$ (**4**, 1 mol%) and $ClMe_2Si$-$SiMe_2Cl$ (**3**, 4 mol%), (112 mg) was reacted with 8.1 mg LiH (50 mol%, in relation to the chlorine content in the disilane mixture) in diglyme in a sealed NMR tube. Cleavage of disilanes and reduction of chloromono- and disilanes started at r.t. as indicated by self-warming of the reaction mixture. The products formed after heating the sample to 60 °C (15 min) are listed in Table S7. The cleavage of disilanes was nearly quantitative, only highly methylated disilane **3** remained in traces (~1%), monosilane **9** was the main product followed by methylchlorosilane **8**. The overall yield of bifunctional monosilanes was 63%.

Table S7

No.	Compound	mol%
**8**	$MeSiHCl_2$	21
**12**	Me_2SiHCl	9
**9**	$MeSiH_2Cl$	33
**11**	Me_2SiCl_2	19
**14**	Me_3SiCl	2
**7**	$MeSiCl_3$	2
**10**	$MeSiH_3$	13
**3**	$ClMe_2Si\text{-}SiMe_2Cl$	1

5.2. Reactions of highly chlorinated disilanes with LiH (41 mol%)

Product formation in cleavage reactions of the methylchlorodisilane mixture of the sample described in 5.1 with LiH in diglyme can easily be controlled by the amount of LiH reacted. When the disilane mixture (183 mg) was reacted with LiH (41 mol%,10 mg) to avoid formation of the low boiling monosilane **10**, dichlorosilane **8** and **11** became the main products. The overall product composition is listed in Table S8, about 2% of the disilanes remained unreacted. The reaction started at r.t. under self-heating of the sample to about 40 °C, the overall yield of bifunctional monosilanes was more than 50%.

Table S8

No.	Compound	mol%
**8**	$MeSiHCl_2$	38
**12**	Me_2SiHCl	7
**9**	$MeSiH_2Cl$	6
**11**	Me_2SiCl_2	34
**14**	Me_3SiCl	2
**7**	$MeSiCl_3$	11
**1**	$Cl_2MeSi\text{-}SiMeCl_2$	<1
**3**	$ClMe_2Si\text{-}SiMe_2Cl$	2

5.3. Reactions of highly chlorinated disilanes with different molar amounts of LiH

The results of Example 5.2 were further supported treating the mixture of disilanes (110 mg – 159 mg), listed in Example 5.1, with different molar amounts of LiH (25, 50, 75, 100 and 400 mol-%, in relation to the chlorine content of the disilane mixture) in diglyme in sealed NMR tubes. All reactions started already at r.t. with self-heating of the samples to about 60 °C. The results of this series of experiments are depicted in Table S9.

Table S9

	**LiH conc. [mol%]**	**25**	**50*)**	**75**	**100**	**400**
**No.**	**Compound**	mol%	mol%	mol%	mol%	mol%
**8**	$MeSiHCl_2$	34	21	6	2	-
**12**	Me_2SiHCl	13	13	12	12	-
**9**	$MeSiH_2Cl$	20	35	30	24	-
**13**	Me_2SiH_2	-	-	2	3	6
**10**	$MeSiH_3$	9	13	41	56	78
**11**	Me_2SiCl_2	21	15	7	2	-
**14**	Me_3SiCl	2	2	1	traces	-
**3**	$ClMe_2Si\text{-}SiMe_2Cl$	1	1	1	1	-
**16**	$H_2MeSi\text{-}SiMeH_2$	-	-	-	traces	8
**17**	$H_2MeSi\text{-}SiMe_2H$	-	-	-	-	5
**19**	$Me_3Si\text{-}SiMeH_2$	-	-	-	-	2

**18**	$HMe_2Si\text{-}SiMe_2H$	-	-	-	-	1

*): This experiment was performed to prove reproducibility of Example 3.1

In summary, for a preferred synthesis of monohydrido substituted silanes such as **8** and **12**, LiH should be used in stoichiometric deficit (< about 25 mol%). For an increase of the amount of monosilane **9** the molar amount of LiH is best between 50 and 75 mol%. For a complete formation of perhydrido-methylsilanes (**10** and **13**) LiH should be used in excess. ^{29}Si NMR spectra of the samples reacted with different molar amounts of LiH are exemplarily shown in Figure S6.

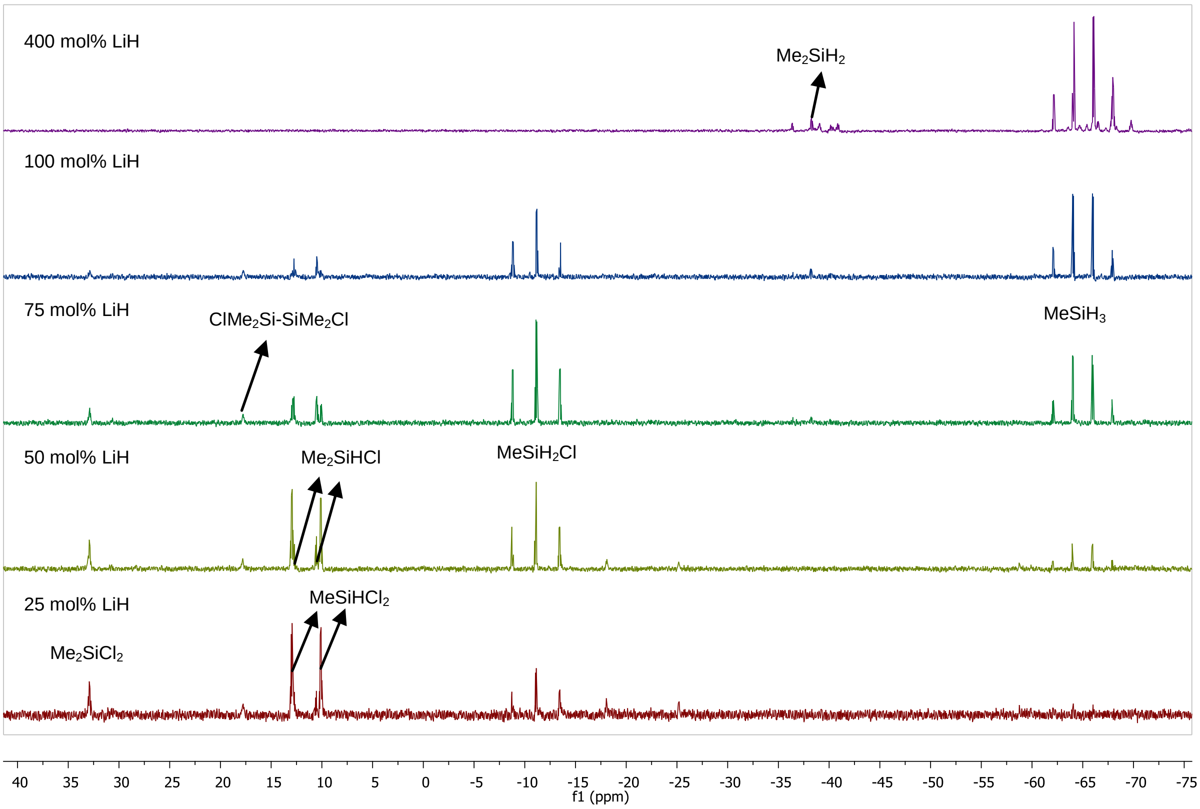

Figure S6: ^{29}Si NMR spectra of samples running disilane cleavage reactions with different molar amounts of LiH.

5.4. Reactions of highly methylated disilanes with different molar amounts of LiH

A mixture (96 mg) of highly methylated disilanes **3** (61 mol%) and **5** (30 mol%) admixed with traces of **2** (5 mol%) and **4** (4 mol%) was reacted with different molar ratios of LiH in diglyme at different reaction temperatures. Reactions were performed in sealed NMR tubes to avoid evaporation of low boiling silane monomers. While highly chlorinated disilanes started to cleave already at r.t. with self-heating of the sample, highly methylated disilanes did not react under comparable conditions. Instead, chlorodisilane hydrogenation slowly started at r.t. and was improved with increasing temperature and the amount of LiH reacted (Table S10). Disilane cleavage slowly started at 140 °C with 77 mol% LiH and monosilanes **13** and **15** were formed in small amounts. Even with a small excess of LiH (110 mol%) monosilane formation is only 8 mol%, with 350 mol% LiH the chlorinated disilanes were completely transformed into their hydrogen

substituted congeners at r.t.. At 140 °C Me_2SiH_2 was formed in 42%, Me_3SiH was 3%. As can be seen from Table S10, the disilanes Me_3Si-$SiMe_2H$ and HMe_2Si-$SiMe_2H$ were slowly cleaved into monomers, the latter faster than pentamethyldisilane.

Table S10

	LiH conc. [mol%]	44	44	77	77	110	110	350	350
	**Temperature [°C]**	**r.t.**	**140**	**r.t.**	**140**	**r.t.**	**140**	**r.t.**	**140**
**No.**	**Compound**	mol%	mol%	mol%	mol%	mol%	mol%	mol%	mol%
**3**	$ClMe_2Si-SiMe_2Cl$	12	7	10	-	3	-	-	-
**5**	$Me_3Si-SiMe_2Cl$	22	19	22	-	14	-	-	-
**21**	$ClMe_2Si-SiMe_2H$	14	11	11	-	8	-	-	-
**20**	$Me_3Si-SiMe_2H$	7	10	10	28	15	28	30	24
**18**	$HMe_2Si-SiMe_2H$	45	53	47	69	60	64	65	31
**13**	Me_2SiH_2	-	-	-	1		3	3	42
**15**	Me_3SiH	-	-	-	2		5	2	3

5.5. Reactions of highly chlorinated disilanes with LiH (41 and 73 mol%)

The reaction of a complex mixture (131 – 238 mg) of mainly highly chlorinated disilanes and monosilanes as displayed in Table S11 was reacted with 41 mol% or 73 mol% LiH, respectively, in diglyme at r.t. in sealed NMR tubes and started with self-heating of the sample. The products formed are listed in Table S12 and prove that monosilanes were formed in 96%, 4% of tetramethyldichloro- and pentamethylchlorodisilane remained unreacted. Higher amounts of LiH led to increasing amounts of hydrogen substituted monosilanes by Si-Cl→Si-H reduction. While the overall yield of bifunctional monosilanes was ~50% using 41mol% of LiH, it was 58% with 73 mol% of LiH, with methylchlorosilane (**9**) being the main cleavage product.

Table S11

No.	Starting mixture	mol%
**11**	Me_2SiCl_2	8
**5**	$Me_3Si-SiMe_2Cl$	2
**3**	$ClMe_2Si-SiMe_2Cl$	3
**1**	$Cl_2MeSi-SiMeCl_2$	50
**2**	$ClMe_2Si-SiMeCl_2$	34
**4**	$Me_3Si-SiMeCl_2$	3
**6**	$Me_3Si-SiMe_3$	traces

Table S12

	LiH conc. [mol%]	41	73
**No.**	**Compound**	mol%	mol%
**8**	$MeSiHCl_2$	17	11
**12**	Me_2SiHCl	14	21
**9**	$MeSiH_2Cl$	20	26
**13**	Me_2SiH_2	-	2
**10**	$MeSiH_3$	6	16
**11**	Me_2SiCl_2	35	16
**14**	Me_3SiCl	4	4
**3**	$ClMe_2Si-SiMe_2Cl$	2	2
**5**	$Me_3Si-SiMe_2Cl$	2	2
**6**	$Me_3Si-SiMe_3$	traces	traces

5.6. Reactions of a mixture of highly methylated disilanes and different carbodisilanes, representing conventionally uncleavable components of the DPR, with LiH (50 and 100 mol%)

A complex mixture (149 – 166 mg) of chlorinated monosilanes, disilanes and carbodisilanes as displayed in Table S13 was reacted with LiH (50 and 100 mol%, respectively) in diglyme at r.t. and at 140 °C (100 mol% LiH) in sealed NMR tubes. Products formed are listed in Table S14 and demonstrate that at 140 °C even the highly chlorinated carbodisilanes $(Cl_2MeSi)_2CH_2$ (**30**) and $ClMe_2Si-CH_2-SiMeCl_2$ (**31**) were cleaved mainly giving monosilane **10**. Only 2% of pentamethyldisilane remained uncleaved.

Table S13

No.	Compound	mol%
**11**	Me_2SiCl_2	7
**14**	Me_3SiCl	7
**7**	$MeSiCl_3$	13
**12**	Me_2SiHCl	4
**8**	$MeSiHCl_2$	2
**5**	$Me_3Si\text{-}SiMe_2Cl$	4
**3**	$ClMe_2Si\text{-}SiMe_2Cl$	18
**1**	$Cl_2MeSi\text{-}SiMeCl_2$	1
**6**	$Me_3Si\text{-}SiMe_3$	<1
**34**	$Me_3Si\text{-}CH_2\text{-}SiMe_2Cl$	4
**32**	$ClMe_2Si\text{-}CH_2\text{-}SiMe_2Cl$	5
**30**	$Cl_2MeSi\text{-}CH_2\text{-}SiMeCl_2$	14
**31**	$ClMe_2Si\text{-}CH_2\text{-}SiMeCl_2$	11
**33**	$Me_3Si\text{-}CH_2\text{-}SiMeCl_2$	2
**35**	$Me_3Si\text{-}CH_2\text{-}SiMe_3$	8

Table S14

	**LiH conc. [mol%]**	**50**	**100**	**100**
	**Temperature [°C]**	**r.t.**	**r.t.**	**140**
**No.**	**Compound**	mol%	mol%	mol%
**11**	Me_2SiCl_2	5	5	-
**14**	Me_3SiCl	6	-	-
**8**	$MeSiHCl_2$	2	-	-
**9**	$MeSiH_2Cl$	5	-	-
**13**	Me_2SiH_2	7	7	17
**10**	$MeSiH_3$	44	75	78
**7**	$MeSiCl_3$	3	-	-
**15**	Me_3SiH	-	-	3
**5**	$Me_3Si\text{-}SiMe_2Cl$	4	-	-
**20**	$Me_3Si\text{-}SiMe_2H$	-	-	2
**21**	$HMe_2Si\text{-}SiMe_2Cl$	11	8	-
**18**	$HMe_2Si\text{-}SiMe_2H$	2	1	-
**16**	$H_2MeSi\text{-}SiMeH_2$	1	-	-
**3**	$ClMe_2Si\text{-}SiMe_2Cl$	8	-	-
	Carbodisilanes	2	4	traces

5.7. Reactions of highly chlorinated disilanes with sodium hydride (50 mol%)

A mixture of highly chlorinated disilanes (122 mg) as depicted in 5.1 was reacted with 50 mol% of sodium hydride (NaH, in relation to chlorine content in the mixture) in diglyme. At r.t. and 60 °C (1 h) no reaction was detected. Heating the sample to 140 °C (30 h) initiated cleavage reactions to yield monosilanes in 53%. Products formed are listed in in Table S15. 47 mol% of the disilanes remained unreacted.

Table S15

No.	Compound	mol%
**8**	$MeSiHCl_2$	4
**9**	$MeSiH_2Cl$	17
**13**	Me_2SiH_2	3
**10**	$MeSiH_3$	2
**11**	Me_2SiCl_2	15
**14**	Me_3SiCl	1
**7**	$MeSiCl_3$	11
**1**	$Cl_2MeSi\text{-}SiMeCl_2$	29
**3**	$ClMe_2Si\text{-}SiMe_2Cl$	4
**2**	$ClMe_2Si\text{-}SiMeCl_2$	14

5.8. Reactions of highly chlorinated disilanes with calcium hydride (50 mol%)

The disilane mixture (244 mg) listed in 5.1 was reacted with 50 mol% of CaH_2 (molar amount of hydride in relation to chlorine content in the mixture) in diglyme. Reaction started at 140 °C (30 h). About 92% of monosilanes were formed and 8% of disilanes remained unreacted. The product composition is given in Table S16.

Table S16

No.	Compound	mol%
**8**	$MeSiHCl_2$	12
**9**	$MeSiH_2Cl$	traces
**11**	Me_2SiCl_2	41
**14**	Me_3SiCl	2
**7**	$MeSiCl_3$	37
**1**	$Cl_2MeSi\text{-}SiMeCl_2$	6
**3**	$ClMe_2Si\text{-}SiMe_2Cl$	2

6. Reactions of different carbodisilanes with LiH

A mixture of different carbodisilanes (234 mg) listed in Table S17 was reacted with lithium hydride (35 mg, 137 mol% in relation to the chlorine content present in the silane mixture) in diglyme as solvent at 180°C (23 h) in a sealed NMR tube to give monomers in 68% (listed in Table S18). Remaining carbodisilanes were fully reduced by excess of lithium hydride present in the mixture (Figure S7).

Table S17

No.	Starting mixture	mol%
**34**	Me_3Si-CH_2-$SiMe_2Cl$	10
**32**	$ClMe_2Si$-CH_2-$SiMe_2Cl$	14
**30**	Cl_2MeSi-CH_2-$SiMeCl_2$	45
**31**	$ClMe_2Si$-CH_2-$SiMeCl_2$	31
**35**	Me_3Si-CH_2-$SiMe_3$	<1

Table S18

No.	Compound	mol%
**36**	HMe_2Si-CH_2-$SiMe_3$	2
**37**	HMe_2Si-CH_2-$SiMe_2H$	5
**38**	H_2MeSi-CH_2-$SiMe_2H$	10
**39**	H_2MeSi-CH_2-$SiMeH_2$	15.0
**13**	Me_2SiH_2	31
**10**	$MeSiH_3$	37

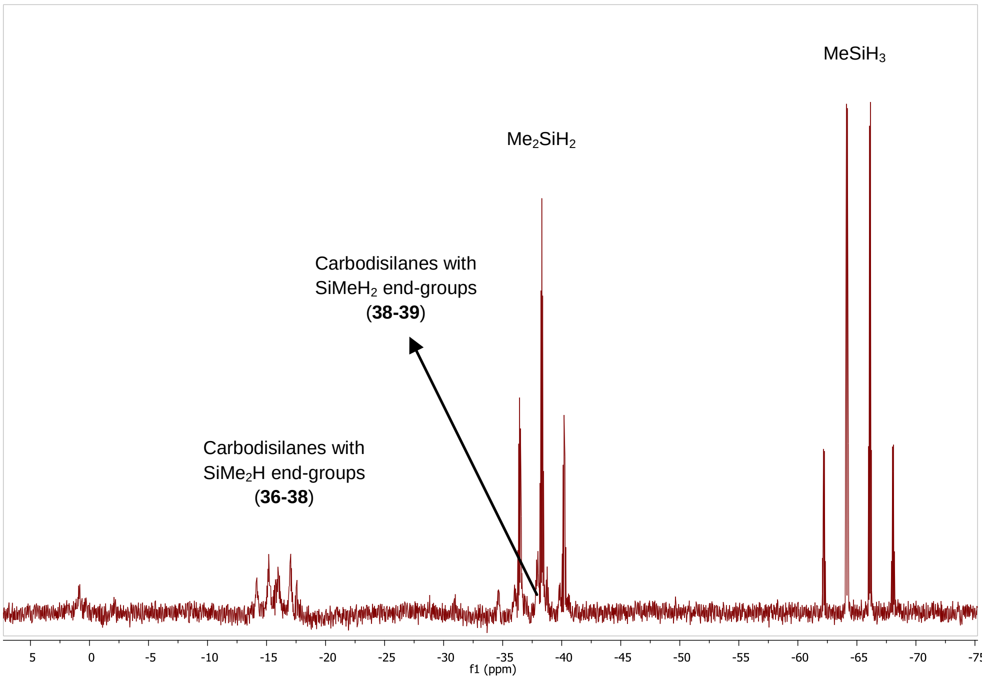

Figure S7: ^{29}Si NMR spectrum of the sample after heating to 180 °C.

Further investigations on the cleavage reactions of chlorinated carbodisilanes with lithium chloride showed that the chloride salt did not act as a cleavage catalyst, $MeSiCl_3$ (**7**) was formed only in traces.

7. Reduction and cleavage reactions of different disilanes with LiH in open systems

7.1. General

Hydrogenation and cleavage reactions of complex mixtures of mono- and disilanes with different molar amounts of LiH in diglyme were performed in open systems to principally show the upscaling procedure: Thus, a 500 mL three-necked flask, equipped with a reflux condenser, a dropping funnel, thermometer and magnetic stirrer, was evacuated and filled with nitrogen to create an inert atmosphere. A cooled trap (-196 °C) was connected with the top of the reflux condenser to collect low boiling monosilanes that evaporate from the reaction mixture under normal pressure.

7.2. Reactions of a mixture of mono- and disilanes with LiH (44 mol%)

14.98 g of a mixture consisting of mono- and disilanes as displayed in Table S19 (together 0.071 mol) were added to the LiH/diglyme suspension (820 mg, 103 mmol, 44 mol% of LiH suspended in 20 mL diglyme) and then carefully heated to 100-130 °C over a period of 5.5 h. The reaction products formed were evaporated, collected in the cold trap and subsequently condensed into an ampule (-196 °C) under vacuo upon warming the cold trap to r.t.. The volatile silanes obtained (5.73 g, together 0.056 mol) are listed in Table S20: The methylchlorosilanes **8** (36%), **9** (15%) and methylsilane **10** (15%) were formed as major products along with **11** (17%).

The residue, still dissolved in diglyme, consisted of monosilanes and highly methylated disilanes **3** and **5** that can´t be cleaved by LiH under moderate conditions. The molar ratio of products remaining is depicted in Table S21.

Tables S19-S21 convincingly prove Si-Si bond cleavage of methylchlorodisilanes with more than one chlorine substituent at the silicon backbone and subsequent reduction of chlorinated monosilanes to yield hydridosilanes under moderate conditions.

Table S19

No.	Starting mixture	mol%	Weight [g]
**11**	Me_2SiCl_2	8	0.73
**5**	Me_3Si-$SiMe_2Cl$	2	0.24
**3**	$ClMe_2Si$-$SiMe_2Cl$	3	0.46
**1**	Cl_2MeSi-$SiMeCl_2$	50	8.09
**2**	$ClMe_2Si$-$SiMeCl_2$	34	5.01
**4**	Me_3Si-$SiMeCl_2$	3	0.40
**6**	Me_3Si-$SiMe_3$	<1	0.05
		**Total:**	**14.98**

Table S20

No.	Compound	mol%	Weight [g]
**11**	Me_2SiCl_2	17	1.21
**14**	Me_3SiCl	3	0.18
**7**	$MeSiCl_3$	6	0.50
**12**	Me_2SiHCl	6	0.32
**8**	$MeSiHCl_2$	36	2.35
**9**	$MeSiH_2Cl$	15	0.70
**13**	Me_2SiH_2	2	0.07
**10**	$MeSiH_3$	15	0.40
		**Total:**	**5.73**

Table S21

No.	Compound	mol%
**11**	Me_2SiCl_2	57
**14**	Me_3SiCl	3
**7**	$MeSiCl_3$	9
**12**	Me_2SiHCl	6
**8**	$MeSiHCl_2$	14
**5**	Me_3Si-$SiMe_2Cl$	4
**3**	$ClMe_2Si$-$SiMe_2Cl$	7

7.3. Reactions of a mixture of mono- and disilanes with LiH (84 mol%)

Similarly to Example 7.2, 10.62 g of a mixture of mono- and disilanes (0.06 mol) as displayed in Table S22 were reacted with 0.92 g (0.112 mol, 84 mol%) of LiH suspended in 6 mL diglyme to give the products listed in Table S23. Products were collected in the -196 °C cooling trap and subsequently condensed in vacuo into an ampule. The reaction time was 5 h, the reaction temperature was 130 °C.

As can be seen from Table S23, 2.69 g of different silane compounds (0.03 mol) were obtained consisting of $MeSiH_3$ (**10**) as the main product (~34%) followed by **7** with 11% and Me_2SiH_2 (**13**) in about 10%. According to the relatively high molar amount of LiH, cleavage of highly chlorinated disilanes and reduction to give methylhydridodisilanes occurred simultaneously. About 7% of per hydrogenated methyldisilanes remained uncleaved, along with permethylated disilane **6** (3%) and dichlorinated disilane **3** (in traces, 1%). The reaction residue mainly contained disilanes as well as carbodisilanes, as depicted in Table S24.

Table S22

No.	Starting mixture	mol%	Weight [g]
**11**	Me_2SiCl_2	12	0.96
**14**	Me_3SiCl	2	0.14
**7**	$MeSiCl_3$	12	1.03
**5**	Me_3Si-$SiMe_2Cl$	17	1.68
**3**	$ClMe_2Si$-$SiMe_2Cl$	23	2.53
**1**	Cl_2MeSi-$SiMeCl_2$	16	2.20
**2**	$ClMe_2Si$-$SiMeCl_2$	7	0.88
**4**	Me_3Si-$SiMeCl_2$	3	0.30
**6**	Me_3Si-$SiMe_3$	3	0.21
**34**	Me_3Si-CH_2-$SiMe_2Cl$	3	0.33
**30**	Cl_2MeSi-CH_2-$SiMeCl_2$	<1	0.06
**31**	$ClMe_2Si$-CH_2-$SiMeCl_2$	1	0.12
**33**	Me_3Si-CH_2-$SiMeCl_2$	<1	0.05
**35**	Me_3Si-CH_2-$SiMe_3$	1	0.13
		**Total:**	**10.62**

Table S23

No.	Compound	mol%	Weight [g]
**11**	Me_2SiCl_2	16	0.60
**14**	Me_3SiCl	1	0.04
**7**	$MeSiCl_3$	11	0.48
**12**	Me_2SiHCl	3	0.07
**8**	$MeSiHCl_2$	4	0.12
**9**	$MeSiH_2Cl$	2	0.04
**13**	Me_2SiH_2	10	0.17
**10**	$MeSiH_3$	34	0.43
**15**	Me_3SiH	1	0.02
**5**	$Me_3Si\text{-}SiMe_2Cl$	6	0.30
**3**	$ClMe_2Si\text{-}SiMe_2Cl$	1	0.06
**6**	$Me_3Si\text{-}SiMe_3$	3	0.11
**20**	$Me_3Si\text{-}SiMe_2H$	1	0.03
**18**	$HMe_2Si\text{-}SiMe_2H$	1	0.07
**16**	$H_2MeSi\text{-}SiMeH_2$	4	0.10
**17**	$HMe_2Si\text{-}SiMeH_2$	1	0.02
**19**	$Me_3Si\text{-}SiMeH_2$	<1	0.03
		**Total:**	**2.69**

Table S24

No.	Compound	mol%
**5**	$Me_3Si\text{-}SiMe_2Cl$	46
**3**	$ClMe_2Si\text{-}SiMe_2Cl$	32
**18**	$HMe_2Si\text{-}SiMe_2H$	5
**21**	$ClMe_2Si\text{-}SiMe_2H$	4
**6**	$Me_3Si\text{-}SiMe_3$	3
	Carbodisilanes	10

7.4. Reactions of a mixture of mono-, di- and carbodisilanes with LiH (43 mol%)

110.9 g of a mixture containing mono-, carbo- and disilanes (together 0.58 mol) which reflects an industrial DPR (see Table S25) were reacted with 9.15 g (1.15 mol, 43 mol%) of LiH in 60 mL diglyme at 130 °C for 5 h. The obtained volatile products that evaporated into the -196 °C cooling trap are listed in Table S26 and prove Me_2SiCl_2 (**11**, 36%), $MeSiCl_3$ (**7**, 19%) and $MeSiHCl_2$ (**8**, 16%) to be main products with nearly 71% (together 0.25 mol). For improved yields of technically most valuable monosilanes, and simplification of work up, the low boiling monosilanes **10** and **13** were directly evaporated into a HCl/diglyme solution to finally give Me_2SiHCl (**12**, 36%), $MeSiH_2Cl$ (**9**, 49%) and $MeSiHCl_2$ (**8**, 8%) in nearly 93%, as displayed in Table S27 (together 0.02 mol). In the reaction residue only disilanes **3** (33%) and **5** (56%) besides carbodisilanes (11%) were detected by ^{29}Si NMR spectroscopy of the sample, as can be seen from Table S28.

Table S25

No.	Starting mixture	mol%	Weight [g]
**11**	Me_2SiCl_2	9	6.46
**14**	Me_3SiCl	3	1.81
**7**	$MeSiCl_3$	7	6.24
**12**	Me_2SiHCl	1	0.53
**8**	$MeSiHCl_2$	1	0.64
**5**	Me_3Si-$SiMe_2Cl$	7	6.50
**3**	$ClMe_2Si$ $SiMe_2Cl$	13	14.59
**1**	Cl_2MeSi-$SiMeCl_2$	24	31.73
**2**	$ClMe_2Si$-$SiMeCl_2$	16	19.07
**4**	Me_3Si-$SiMeCl_2$	2	2.60
**6**	Me_3Si-$SiMe_3$	<1	0.61
**34**	Me_3Si-CH_2-$SiMe_2Cl$	3	3.02
**32**	$ClMe_2Si$-CH_2-$SiMe_2Cl$	2	2.52
**30**	Cl_2MeSi-CH_2-$SiMeCl_2$	5	6.40
**31**	$ClMe_2Si$-CH_2-$SiMeCl_2$	4	4.93
**33**	Me_3Si-CH_2-$SiMeCl_2$	2	2.80
**35**	Me_3Si-CH_2-$SiMe_3$	<1	0.45
		**Total**	**110.9**

Table S26

No.	Compound	mol%	Weight [g]
**11**	Me_2SiCl_2	36	11.38
**14**	Me_3SiCl	3	0.77
**7**	$MeSiCl_3$	19	6.72
**12**	Me_2SiHCl	4	1.00
**8**	$MeSiHCl_2$	16	4.46
**9**	$MeSiH_2Cl$	6	1.28
**13**	Me_2SiH_2	3	0.48
**10**	$MeSiH_3$	1	0.16
**5**	Me_3Si-$SiMe_2Cl$	5	2.06
**3**	$ClMe_2Si$ $SiMe_2Cl$	1	0.66
**18**	HMe_2Si $SiMe_2H$	2	0.74
**16**	H_2MeSi-$SiMeH_2$	1	0.32
**6**	Me_3Si-$SiMe_3$	2	0.65
		**Total:**	**30.68**

Table S27

No.	Compound	mol%	Weight [g]
**11**	Me_2SiCl_2	7	0.19
**12**	Me_2SiHCl	36	0.69
**9**	$MeSiH_2Cl$	49	0.80
**8**	$MeSiHCl_2$	8	0.17
		**Total**	**1.85**

Table S28

No.	Compound	mol%
**5**	Me_3Si-$SiMe_2Cl$	56
**3**	$ClMe_2Si$-$SiMe_2Cl$	33
	Carbodisilane	11

8. Cleavage of a mixture of highly chlorinated disilanes with different alkali and alkaline earth metal chlorides

Metal chlorides (60-170 mg) were suspended in an industrial DPR mixture (0.2 mL, composition listed in Table S29, entry *a*) with dry diglyme (0.4 mL) as solvent in an NMR tube, frozen (-196 °C) evacuated and sealed in vacuo. NMR spectra were taken reacting the samples with increasing reaction temperatures and reaction times. Oligosilanes formed in the disproportionation reactions were not detected by NMR. $MeSiHCl_2$ (**8**) was obviously formed from traces of residual water, released from the salts upon heating the mixtures. Hydrolysis of chlorosilanes subsequently gives hydrogen chloride that acts as hydrogen source to form Si-H bonds. Simultaneously, siloxanes are formed (in traces), in some cases giving insoluble polymers or solids that could not be identified by ^{29}Si NMR spectroscopy.

Table S29: Cleavage of disilanes representing the DPR with different alkali and alkaline earth metal chlorides in different glymes as solvents.*)

Entry	Metal chloride	Solvent	Temperature/°C (time)	$MeSiCl_3$	Me_2SiCl_2	Me_3SiCl	$MeSiHCl_2$**)	Cl_2MeSi-$SiMeCl_2$	$ClMe_2Si$-$SiMeCl_2$	$ClMe_2Si$-$SiMe_2Cl$	Me_3Si-$SiMeCl_2$	Me_3Si-$SiMe_2Cl$
a		**C_6D_6**	**-**	**-**	**9**	**-**	**-**	**48**	**35**	**5**	**1**	**2**
b	LiCl	Diglyme	140 (9 h)	44	44	3	2	2	-	3	-	2
c	NaCl	Diglyme	140 (13 h)<br>180 (10 h)	-<br>7	10<br>48	-<br>4	-<br>-	48<br>22	34<br>13	5<br>2	1<br>1	2<br>2
d	NaCl	Tetraglyme	140 (13 h)<br>180 (10 h)	15<br>32	41<br>47	23<br>2	-<br>7	20<br>5	14<br>2	4<br>3	2<br>-	1<br>1
e	KCl	Diglyme	140 (13 h)<br>180 (10 h)	2<br>14	15<br>32	-<br>1	-<br>-	43<br>30	31<br>17	4<br>5	1<br>1	1<br>1
f	KCl	Tetraglyme	140 (13 h)<br>180 (10 h)	37<br>43	48<br>43	-<br>2	-<br>4	6<br>2	4<br>1	3<br>3	1<br>-	2<br>2
g	$CaCl_2$	Diglyme	140 (13 h)<br>180 (10 h)	3<br>27	14<br>48	-<br>4	-<br>6	37<br>7	35<br>3	5<br>3	3<br>1	2<br>2
h	$CaCl_2$	Tetraglyme	140 (13 h)<br>180 (10 h)	17<br>35	40<br>46	4<br>2	2<br>5	20<br>4	9<br>2	4<br>3	2<br>1	2<br>2
j	$MgCl_2$	Diglyme	140 (13 h)<br>180 (10 h)	4<br>21	21<br>45	2<br>4	8<br>11	34<br>9	24<br>4	5<br>3	1<br>1	2<br>1
k	$MgCl_2$	Tetraglyme	140 (13 h)<br>180 (10 h)	35<br>35	43<br>41	1<br>2	12<br>15	3<br>2	-<br>-	2<br>3	-<br>-	2<br>2

*) Product yields in mol %; **) $MeSiHCl_2$ most likely results from reaction with residual water present in the metal salts.

9. Cleavage reactions of highly branched oligosilanes with LiH

Highly branched oligosilanes with Cl_2MeSi- end groups (^{29}Si-NMR spectrum is depicted in Figure S8 (red). This oligomers were obtained after treatment of a disilane mixture (listed in Table S11) with 1-methylimidazole and a reaction time of 1 week[1a]) dissolved in C_6D_6 (0.35ml) were reacted with LiH (35 mg) in diglyme as solvent in a sealed NMR tube. Reactions were performed at 180 °C for 20 h and 220°C for 24 h. Heating the sample to T>180°C showed significant cleavage of the highly branched oligosilanes to yield products listed in Table S30.

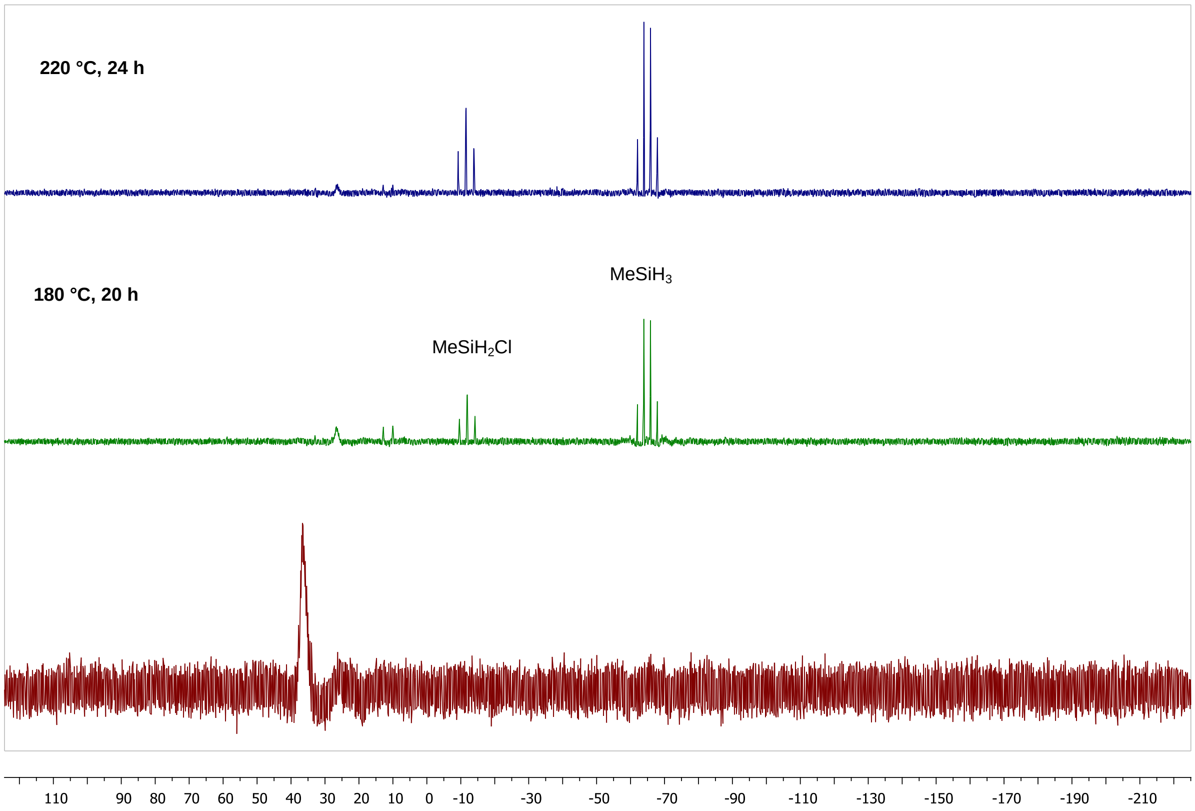

Figure S8: ^{29}Si NMR spectrum of highly branched oligosilanes with the broad signal at δ^{29}Si= +35 ppm reacted with LiH at 180 °C (green) and 220 °C (blue)

Table S30

No.	Compound	180 °C mol%	220 °C mol%
**12**	Me_2SiHCl	-	1
**8**	$MeSiHCl_2$	8	3
**9**	$MeSiH_2Cl$	28	28
**13**	Me_2SiH_2	-	1
**10**	$MeSiH_3$	31	52
	polymer	33	15

10. Cleavage reactions of highly branched oligosilanes with LiCl

Highly branched oligosilanes with Cl_2MeSi- end groups (see chapter 9.), dissolved in C_6D_6 (0.35ml), were reacted with LiCl (91 mg) in diglyme in a sealed NMR tube. Reactions were performed at 180 °C for 20 h and 220°C for 52 h. Heating the sample to 180°C showed no significant cleavage of the oligosilanes. Only at higher temperatures (220 °C) significant cleavage of the oligosilanes was observed NMR spectroscopically to yield oligosilanes and monosilanes, such as Me_2SiCl_2 and $MeSiCl_3$, in a molar ratio of 1:1.

11. Cleavage reactions of highly chlorinated disilanes with lithium and potassium chloride in open systems

11.1. Disilane cleavage reactions with LiCl

11.91 g of a mixture comprising of mono- and disilanes as displayed in Table S11 were placed in a flask equipped with a condenser, and 2.88 g of LiCl (68 mmol) were added and suspended in 10 mL of diglyme. The reaction mixture was heated to 145 °C for 5.5 h. After cooling to r.t. the reaction products formed were evaporated and collected in a cold trap (-196°C) and subsequently condensed under vacuo into an ampule (-196 °C) upon warming the cold trap to r.t.. The silane mixture obtained is listed in Table S31 and demonstrates that the cleavage of highly chlorinated disilanes can be carried out in a preparative scale to give methylchlorosilanes **7** and **11** nearly quantitatively. The residue consists of carbosilanes, highly methylated disilanes and traces of the monosilanes $MeSiCl_3$ and Me_2SiCl_2. Furthermore, oligosilanes, which formed upon disproportionation reaction, were detected in smaller amounts in the ^{29}Si-NMR spectrum.

Table S31

No.	Compound	mol%	Weight [g]
**11**	Me_2SiCl_2	42	2.73
**14**	Me_3SiCl	3	0.16
**7**	$MeSiCl_3$	54	4.02
**5**	$Me_3Si\text{-}SiMe_2Cl$	1	0.07
**3**	$ClMe_2Si\text{-}SiMe_2Cl$	<1	0.04
		**Total:**	**7.02**

11.2. Disilane cleavage reactions with KCl

59.6 g of a mixture comprising of mono- and disilanes as displayed in Table S11 were added to 1.98 g KCl (26 mmol) suspended in 10 mL of diglyme. The reaction mixture was heated to 145 -170 °C for 18 h. The reaction products formed were distilled off and their molar ratio is listed in Table S32. The product composition demonstrates that the cleavage of highly chlorinated disilanes can be carried out in a preparative scale to give methylchlorosilanes **7** and **11** (together 0.2 mol). $MeSiHCl_2$ (**8**, 1%) formation might be explained due to carbodisilane formation.[9] Notably, potassium chloride was used in this disilane cleavage reaction as catalyst (3.3 weight%). In the reaction residue carbosilanes, highly methylated disilanes and traces of the monosilanes $MeSiCl_3$ and Me_2SiCl_2 could be identified besides larger amounts of oligosilanes that were formed upon disproportionation reactions.

Table S32

No.	Compound	mol%	Weight [g]
**11**	Me_2SiCl_2	46	11.76
**14**	Me_3SiCl	2	0.69
**7**	$MeSiCl_3$	51	15.11
**8**	$MeSiHCl_2$	1	0.21
		**Total:**	**27.77**

12. Disilane cleavage reactions of a mixture of highly chlorinated disilanes with LiCl in the presence of a HCl/diglyme solution

A mixture of disilanes (238 mg, listed in Table S19) admixed with 8 % Me_2SiCl_2, was reacted with lithium chloride (101 mg) in a diglyme/HCl solution (molar ratio disilane/HCl 1:1) at 80°C (71 h) to give monomers nearly quantitatively (listed in Table S33). For this reaction only HCl was used to form Si-H bonds. HCl reacts as a silylene trapping agent, thus, the silylene is prevented to insert into Si-Si-bonds to build oligomeric structures. The main products of this very efficient reaction were $MeSiCl_3$ (34%) and $MeSiHCl_2$ (31%).

Table S33

No.	Compound	mol%
**11**	Me_2SiCl_2	28
**14**	Me_3SiCl	2
**7**	$MeSiCl_3$	34
**8**	$MeSiHCl_2$	31
**9**	$MeSiH_2Cl$	2
**1**	Cl_2MeSi-$SiMeCl_2$	1
**2**	$ClMe_2Si$-$SiMeCl_2$	1
**3**	$ClMe_2Si$-$SiMe_2Cl$	1

13. Disilane cleavage reactions of a mixture consisting mainly of highly chlorinated disilanes with KCl in the presence of a HCl/diglyme solution

A mixture of disilanes (238 mg, listed in Table S19), admixed with 8 % of Me_2SiCl_2, was reacted with potassium chloride (95 mg) in a tetraglyme/diglyme/HCl solution (molar ratio disilane/HCl 1:1) at 80°C (71 h) to give monomers in 52% (listed in Table S34). $ClMe_2Si$-$SiMeCl_2$ (**2**) was faster cleaved than Cl_2MeSi-$SiMeCl_2$ (**1**) to give Me_2SiCl_2 (**11**, 38%) and $MeSiHCl_2$ (**8**, 10%). Increasing the temperature to 100 °C (23.5 h) gave monomers in 70% besides the non-cleaved disilanes listed in Table S34. No oligomeric structures were identified. For this reaction only HCl was used to from Si-H bonds. HCl reacted as a silylene trapping agent, the silylene is prevented to insert into Si-Si-bonds to build oligomeric structures. The main products after reaction was completed were Me_2SiCl_2 (43%) and $MeSiHCl_2$ (20%).

Table S34

	Temperature time	80 °C 71 h	100 °C 23.5 h
**No.**	**Compound**	**mol%**	**mol%**
**11**	Me_2SiCl_2	38	43
**14**	Me_3SiCl	4	2
**7**	$MeSiCl_3$	-	5
**8**	$MeSiHCl_2$	10	20
**1**	Cl_2MeSi-$SiMeCl_2$	32	24
**2**	$ClMe_2Si$-$SiMeCl_2$	12	3
**3**	$ClMe_2Si$-$SiMe_2Cl$	4	3

14. Computational details

Geometry optimizations and harmonic frequency calculations were performed with the Gaussian 09 program[10] employing the M062X density functional[11] in combination with the 6-31+G(d,p) split-valence basis set. The SMD polarizable continuum model[12] was employed to account for solvent effects (tetrahydrofuran). To avoid spurious imaginary frequencies caused by integration grid errors, the 'ultrafine' grid option was used throughout.[13]

14.1. Cartesian coordinates

SMD(THF)-M062X/6-31+G(d,p) optimized geometries (total energies in Hartree)

H_2SiMe_2 (C_{2v})
E_{tot} = –370.446698639
G_{corr} (298K) = 0.062933
Si 0.00000 0.00000 0.55068
H -1.19529 0.00000 1.43560
H 1.19529 0.00000 1.43560
C 0.00000 1.54860 -0.51242
H 0.88523 1.57495 -1.15675
H 0.00000 2.45725 0.09771
H -0.88523 1.57495 -1.15675
C 0.00000 -1.54860 -0.51242
H 0.88523 -1.57495 -1.15675
H -0.88523 -1.57495 -1.15675
H 0.00000 -2.45725 0.09771

$[HSiMe_2]^–$ (C_s)

E_{tot} = –369.903555788
G_{corr} (298K) = 0.062933
Si -0.04278 0.73900 0.00000
C -0.04278 -0.54428 1.47653
H 0.71128 -1.33701 1.36631
H -1.02356 -1.03406 1.54731
H 0.13190 -0.05434 2.44340
C -0.04278 -0.54428 -1.47653
H 0.71128 -1.33701 -1.36631
H 0.13190 -0.05434 -2.44340
H -1.02356 -1.03406 -1.54731
H 1.47294 1.03614 0.00000

$HMe_2Si–SiMe_2H$ (C_{2h})

E_{tot} = – 739.717222973
G_{corr} (298K) = 0.129566
Si -0.31801 1.12696 0.00000
H -1.81023 1.18999 0.00000
C 0.31801 2.00918 1.54356
H 1.41289 1.98518 1.57405
H -0.05491 1.54142 2.46040
H 0.00470 3.05890 1.54833
C 0.31801 2.00918 -1.54356
H 1.41289 1.98518 -1.57405
H 0.00470 3.05890 -1.54833
H -0.05491 1.54142 -2.46040
Si 0.31801 -1.12696 0.00000
H 1.81023 -1.18999 0.00000
C -0.31801 -2.00918 -1.54356
H 0.05491 -1.54142 -2.46040
H -1.41289 -1.98518 -1.57405
H -0.00470 -3.05890 -1.54833
C -0.31801 -2.00918 1.54356
H -0.00470 -3.05890 1.54833
H -1.41289 -1.98518 1.57405
H 0.05491 -1.54142 2.46040

$[HMe_2Si–SiMe_2]^–$ (C_1)
E_{tot} = – 739.178732100
G_{corr} (298K) = 0.116729
Si -0.32054 0.95652 0.00000
H -1.83446 0.88706 0.00000
Si 0.72366 -1.15603 0.00000
C -0.29318 -1.93667 -1.48173
H -0.00805 -1.49835 -2.44709
H -1.38146 -1.81756 -1.36975
H -0.09029 -3.01390 -1.55542
C -0.29318 -1.93667 1.48173
H -0.00805 -1.49835 2.44709
H -0.09029 -3.01390 1.55542
H -1.38146 -1.81756 1.36975
C 0.11484 2.02487 -1.51878
H 1.19758 2.19466 -1.56037
H -0.37749 3.00414 -1.48509
H -0.17724 1.53423 -2.45448
C 0.11484 2.02487 1.51878
H 1.19758 2.19466 1.56037
H -0.17724 1.53423 2.45448
H -0.37749 3.00414 1.48509

TS (i983, C_1)
E_{tot} = –1109.59030028
G_{corr} (298K) = 0.195944

Si	2.50225	-0.28806	0.18352
H	3.56914	0.52594	0.87028
Si	0.40807	0.75392	-0.00154
C	0.71988	2.33322	-1.05222
H	1.10110	2.08989	-2.05123
H	1.42503	3.02923	-0.58109
H	-0.23112	2.86842	-1.18842
C	0.01041	1.45152	1.74326
H	0.06216	0.68315	2.52363
H	-1.01518	1.85138	1.74215
H	0.68203	2.26997	2.03099
C	3.19644	-0.71035	-1.53258
H	2.48450	-1.33498	-2.08458
H	4.14282	-1.25884	-1.46222
H	3.37479	0.19281	-2.12665
C	2.37558	-1.90891	1.16330
H	1.67910	-2.59330	0.66499
H	1.99843	-1.73690	2.17778
H	3.34589	-2.41227	1.24544
H	-1.27081	0.48019	-0.49368
H	-4.43427	0.61871	-0.45130
Si	-3.00524	0.10280	-0.34566
C	-2.93909	-0.66080	1.41187
H	-3.61133	-1.52024	1.52609
H	-3.19111	0.06968	2.18933
H	-1.91705	-1.01549	1.61017
C	-3.01052	-1.42690	-1.50337
H	-3.71354	-2.20376	-1.17866
H	-2.00657	-1.87386	-1.51683
H	-3.26133	-1.15767	-2.53624

15. References

[1] a) U. Herzog, R. Richter, E. Brendler, G. Roewer, *J. Organomet. Chem.* **1996**, *507*, 221-228; b) R. Lehnert, M. Höppner, H. Kelling, *Z. Anorg. Allg. Chem.* **1990**, *591*, 209-213; c) H. Marsmann, in *Oxygen-17 and Silicon-29* (Eds.: P. Diehl, E. Fluck, R. Kosfeld), Springer-Verlag, Berlin, Heidelberg, New York, **1981**, pp. 65-235; d) U. Herzog, E. Brendler, G. Roewer, *J. Organomet. Chem.* **1996**, *511*, 85-91; e) M. N. Missaghi, C. M. Downing, M. C. Kung, H. H. Kung, *Organometallics* **2008**, *27*, 6364-6366.

[2] H. Schmölzer, E. Hengge, *J. Organomet. Chem.* **1984**, *260*, 31-39.

[3] U. Herzog, G. Roewer, U. Pätzold, *J. Organomet. Chem.* **1995**, *494*, 143-147.

[4] a) U. Herzog, G. Rheinwald, *J. Organomet. Chem.* **2001**, *628*, 133-143; b) G. Déléris, M. Birot, J. Dunoguès, B. Barbe, M. Petraud, M. Lefort, *J. Organomet. Chem.* **1984**, *266*, 1-8.

[5] ^{29}Si NMR chemical shift sare obtained from experimental results after treatment of carbodisilane mixture with $LiAlH_4$.

[6] V. Semenov, E. Y. Ladilina, N. Cherepennikova, T. Chesnokova, *Russ. J. Appl. Chem.* **2002**, *75*, 127-134.

[7] H. Stueger, G. Fuerpass, T. Mitterfellner, J. Baumgartner, *Organometallics* **2010**, *29*, 618-623.

[8] a) C. Eaborn, P. B. Hitchcock, P. D. Lickiss, *J. Organomet. Chem.* **1983**, *252*, 281-288; b) C. Eaborn, P. D. Lickiss, *J. Organomet. Chem.* **1985**, *294*, 305-313.

[9] R. H. Baney, J. H. Gaul Jr, T. K. Hilty, *Organometallics* **1983**, *2*, 859-864.

[10] M. J. Frisch, H. B. Schlegel, G. W. Trucks, G. E. Scuseria, M. A. Robb, J. R. Cheeseman, G. Scalmani, V. Barone, B. Mennucci, G. A. Petersson, H. Nakatsuji, M. Caricato, X. Li, H. P. Hratchian, A. F. Izmaylov, J. Bloino, G. Zheng, J. L. Sonnenberg, M. Hada, M. Ehara, K. Toyota, R. Fukuda, J. Hasegawa, M. Ishida, T. Nakajima, Y. Honda, O. Kitao, H. Nakai, T. Vreven, J. A. Montgomery, Jr., J. E. Peralta, F. Ogliaro, M. Bearpark, J. J. Heyd, E. Brothers, K. N. Kudin, V. N. Staroverov, R. Kobayashi, J. Normand, K. Raghavachari, A. Rendell, J. C. Burant, S. S. Iyengar, J. Tomasi, M. Cossi, N. Rega, J. M. Millam, M. Klene, J. E. Knox, J. B. Cross, V. Bakken, C. Adamo, J. Jaramillo, R. Gomperts, R. E. Stratmann, O. Yazyev, A. J. Austin, R. Cammi, C. Pomelli, J. W. Ochterski, R. L. Martin, K. Morokuma, V. G. Zakrzewski, G. A. Voth, P. Salvador, J. J. Dannenberg, S. Dapprich, A. D. Daniels, O. Farkas, J. B. Foresman, J. V. Ortiz, J. Cioslowski, D. J. Fox, *Gaussian 09, Revision D.01 Gaussian, Inc., Wallingford CT,* **2013**.

[11] Y. Zhao, D. G. Truhlar, *Theor. Chem. Acc.* **2008**, *120*, 215-241.
[12] A. V. Marenich, C. J. Cramer, D. G. Truhlar, *J. Phys. Chem. B* **2009**, *113*, 6378-6396.
[13] S. E. Wheeler, K. Houk, *J. Chem. Theory Comput.* **2010**, *6*, 395-404.

16. Author Contributions

T.S. (lead, most experimental and spectroscopic work) and A.G.S. conducted the experimental work. K.M.L., T.F., N.A., and M.C.H. designed the concept and supervised the work. T.S., N.A., and M.C.H. wrote the paper (all equal), A.G.S. (supporting).
